# Assessment of climate change impact on landscape tree distribution and sustainability in South Korea using MaxEnt-based modeling

**DOI:** 10.1371/journal.pone.0316393

**Published:** 2025-03-03

**Authors:** Dong-Joo Kim, Na-Yeon Han, Mi Na Choi, Moon-Jeong Jang, Man-Seok Shin, Chang Wan Seo, Do-Hun Lee, Yong Sung Kwon

**Affiliations:** 1 Environmental Impact Assessment, Division of Ecological Assessment, National Institute of Ecology, Seocheon, Republic of Korea; 2 Diagnosis Department 1, Jeil Tree General Hospital, Yeongcheon, Republic of Korea; 3 Department of Environmental Engineering, Kunsan National University, Gunsan, Republic of Korea; 4 Climate Change and Carbon Research, Division of Ecological Assessment, National Institute of Ecology, Seocheon, Republic of Korea; 5 Division of Ecological Research Strategy, Division of Ecological Assessment, National Institute of Ecology, Seocheon, Republic of Korea; Baotou Medical College, CHINA

## Abstract

The rapidly changing climate is impacting species globally at an unprecedented rate, including humans. Consequently, extensive research is being conducted on the impacts of climate change on indigenous and vulnerable species. However, landscape trees, which are cultivated and managed by humans, receive less attention despite their significant role in urban environments. Landscape tree also have specific climatic ranges and environmental requirements, making them susceptible to climate change. In this study, we predicted the future sustainability of three native landscape trees (*Stewartia koreana*, *Betula ermanii*, and *Taxus cuspidata*) using maximum entropy (MaxEnt) models under SSP2-4.5 and SSP5-8.5 climate scenarios. A time-series analysis of suitability was conducted, and the resulting maps were overlaid to classify regions of suitability. The findings indicate a general northward shift in climate suitability and a potential reduction in long-term suitable areas for all three species. Under the SSP2-4.5 scenario, potential suitable area for *S. koreana* increased, while those for *B. ermanii*, *T. cuspidata* decreased by the 2090s. Under the SSP5-8.5 scenario, suitable areas for *S. koreana*, *B. ermanii*, *T. cuspidata* decreased by 33.6%, 98.9%, and 90.1%, respectively. The climate suitability classification (“Sustainable suitability”, “Risk”, “Inflow”, “Lost”, and “Variable” regions) effectively identified areas of sustainability and risk, as well as regions requiring management. A notable decline in “Sustainable suitability” regions, which remained suitable from the present to the 2090s, was observed under the SSP5-8.5 scenario relative to SSP2-4.5. The methods utilized in this study offer valuable insights for future landscape planning and conservation. This research underscores the need for adaptive strategies to mitigate potential economic and ecological impacts of climate change by utilizing species distribution models for sustainable landscape planning and tree conservation.

## 1. Introduction

According to the Intergovernmental Panel on Climate Change (IPCC)’s Sixth Assessment Report (AR6), global surface temperature increased by 1.09 °C (0.95–1.2 °C) between 1850–1900 and 2011–2020, with this rise being more pronounced over land than over oceans. Furthermore, the rate of temperature rise has accelerated since 1970 [1]. Such drastic environmental changes have led to an increase in vulnerable species unable to adapt, resulting in extinctions [2]. Additionally, Warren et al. [3] predicted that a 2 °C rise in global temperature could result in a 16% reduction in plant distribution. Plant growth is influenced by climate [4,5], and such changes are expected to significantly impact both native species and landscape trees. In South Korea, climate change-mediated rising temperatures are causing a nationwide shift in climatic zones [6]. Forecasting the distribution of species and potential future shifts in suitable habitats due to climate change is essential for protecting ecosystems and for the planning, introduction, cultivation, management, and utilization of new plant species [7–9].

In recent years, various species distribution models (SDMs) such as maximum entropy (MaxEnt) model, generalized linear models, random forests, generalized additive models, generalized boosted trees, and multivariate adaptive regression splines have been widely adopted to explain and predict species distribution trends [10–14]. SDMs utilize presence-absence data, environmental variables, and modeling algorithms. Their greatest advantage lies in their ability to construct desired datasets, allowing researchers to predict the distribution of species [15–17]. Over the past two decades (1999–2019), more than 50% of the approximately 6,000 published documents related to SDMs have focused on assessing the impact of climate change on biodiversity, habitat restoration, and species migration [18]. These efforts to objectively clarify changes in species distribution and their driving factors have subsequently contributed to the development of various conservation policies [19].

MaxEnt is the most popular SDM due to its excellent performance, even with small sample sizes of presence data [20–22]. Santini et al. [10] reported approximately 78% of research between 2015–2019 adopted MaxEnt to predict species distribution. Its reliability has been consistently proven in numerous studies, making it one of the most widely used SDMs today [ 11,23,24]. However, the optimization of MaxEnt models is often overlooked. In almost all cases, especially for complex machine learning models, the default settings are used to develop the model [25]. This practice can reduce model transferability and decrease accuracy during projections [26]. By applying optimization tool such as ENMeval, researchers can determine the optimal parameters for specific input data and assess performance, thereby preventing overfitting and enhancing MaxEnt’s effectiveness [27]. In the field of ecology in South Korea, where species occurrence data are predominantly presence-only [28], MaxEnt is the preferred choice for predicting future climate suitability.

However, most of the previous studies utilizing SDMs have primarily focused on native and vulnerable species unaffected by human activity [29–32]. Landscape trees, subject to human management, are often perceived as less vulnerable to climate change [33], leading to a scarcity of research in this area. Studies that utilize SDMs to make predictions typically focus on short-term, mid-term, or specific future periods [33–37]. Unlike native or vulnerable species, the planting time and location of landscape trees are entirely determined by humans, making them newly introduced. Therefore, selecting and introducing landscape trees with climate change in mind requires monitoring changes at shorter intervals and assessing their long-term sustainability. *Stewartia koreana* and *Betula ermanii* are used as landscape trees and have been designated by South Korean government agencies as Climate-sensitive Biological Indicator Species (CBIS), necessitating ongoing monitoring and management due to climate change [38]. On the other hand, *Taxus cuspidata* is widely cultivated as an ornamental and garden tree, valued for its bark’s exceptional medicinal properties as an anticancer agent [39]. However, its future distribution is anticipated to shrink due to climate change [40]. Hence, landscape trees, sharing the same climatic conditions as native species, are inevitably affected by climate change. Notably, landscape trees offer significant economic benefits [41] that outweigh the cost of planting, similar to wild trees. However, if planted landscape trees cannot be sustained due to uncontrollable climate conditions, it could result in considerable economic loss. Moreover, it is crucial to recognize that research in ecology and economics follows distinct paths [42]. For instance, landscape ecology typically focuses on the interplay between patterns and processes, with limited attention to valuation studies. Conversely, ecological economics tend to overlook the connection between value-generating ecological functions of landscape trees and their physical underpinnings.

This study aims to evaluate and identify sustainable suitability areas and their changes under two different climate change scenarios for landscape trees, which were previously considered to be minimally impacted by climate change. By doing so, this research seeks to provide a comprehensive understanding of how climate change may alter the distribution and suitability of these trees, thereby informing future landscape planning and conservation efforts.

## 2. Materials and methods

### 2.1. Species occurrence data

In this study, we aimed to evaluate the climate suitability of landscape tree species that are simultaneously native species, under projected future climate change scenarios. Three target landscape tree species were selected for climatic regions and usage trends. Initially, 6 landscape tree species, comprising 5 southern and 1 northern species, were screened by referring to the CBIS announced by the Ministry of Environment and National Institute of Biological Resources of Korea. Among the 5 southern species, 4 species (*Neolitsea sericea*, *Melia azedarach*, *Daphniphyllum macropodum*, and *Machilus thunbergii*) were found to primarily inhabit southern regions, with little observed change over time based on data from the 2nd to the 5th National Ecosystem Surveys (1997–2019) S1 Fig. However, the *S. koreana* despite being classified as a southern species, was newly observed in central and northern regions during the 4th and 5th surveys (2014–2019). This was deemed a suitable example for observing changes caused by climate change and was therefore selected as a target species for the study. For the northern species, *B. ermanii* is the only landscape tree species within CBIS. In addition to this, *T. cuspidata* is selected due to its high preference in Korean landscaping projects, high production volume, and recent research statistics [43].

**Fig 1:**
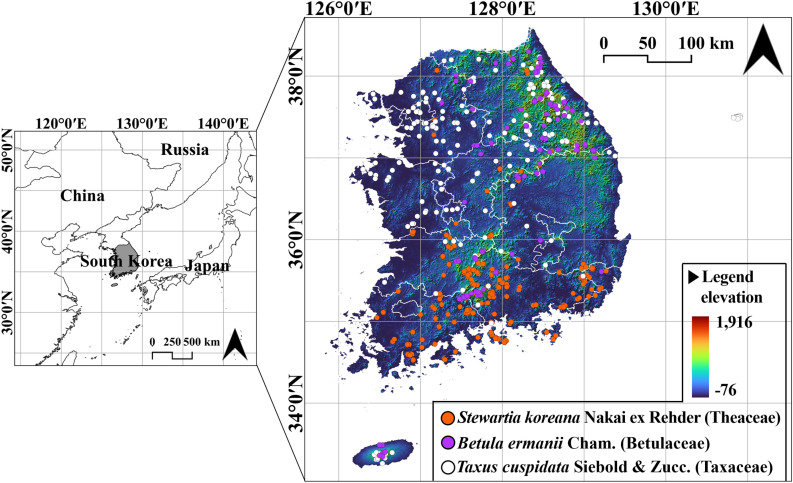
Occurrence map of *Stewartia koreana* Nakai ex Rehder (Theaceae)*, Betula ermanii* Cham. (Betulaceae), and *Taxus cuspidata* Siebold & Zucc. (Taxaceae) in South Korea.

Occurrence records for the selected species were collected from 2006 to 2019 for three species: 187 for *S. koreana,* 109 for *B. ermanii*, and 218 for *T. cuspidata*. The occurrence map of the three species is represented in Fig 1. The data were sourced from (1) the 3rd, 4th, and 5th National Ecosystem Surveys [44], and (2) the GBIF biodiversity data [45].

### 2.2. Bioclimatic variables

Climate change is perceived as a risk across various fields [46–48], and climate change scenarios are a crucial method for exploring the uncertainties of future climates [49,50]. In this study, climate change was likewise approached from the perspective of risk for landscape trees. The recently adopted Shared Socioeconomic Pathways (SSP) scenarios provide several options, and it was determined that considering more high-risk scenarios is necessary to prepare for future uncertainties and risks. The SSP1 scenario, which assumes the successful achievement of climate change mitigation and sustainable development, was excluded as it was deemed an overly optimistic approach. Although the SSP3 and SSP4 scenarios consider detailed assumptions such as demographic and economic developments, this study focused on assessing the potential impacts of climate change on landscape trees. Therefore, we adopted two scenarios: SSP2, which assumes intermediate levels of all factors, and SSP5, which represents an extreme pathway.

The bioclimatic data for South Korea, including records of maximum temperature, minimum temperature, and precipitation, were obtained from the Korea Meteorological Administration [51] with a spatial resolution of 1 km. Using the “biovars” function in the R [52] package “dismo”, 19 bioclimatic variables were generated. This study utilized bioclimatic data from 2000–2019, corresponding to the period during which occurrence records were collected, to project future climate scenarios for the following decades: 2020s (2000–2030), 2030s (2021–2040), 2040s (2031–2050), 2050s (2041–2060), 2060s (2051–2070), 2070s (2061–2080), 2080s (2071–2090), and 2090s (2081–2100). Present and projected climate scenarios utilized the 5ENSMN ensemble model developed by the Korea Meteorological Administration for a detailed projection of South Korea’s climate. This model incorporates an ensemble of five General Circulation Models: HadGEM3-RA, WRF, CCLM, GRIMs, and RegCM4. Additionally, it includes shared socioeconomic pathway (SSPs) scenarios, as adopted in the AR6 published by the IPCC. To assess the future climate suitability for *S. koreana, B. ermanii*, and *T. cuspidata*, we focused our analysis exclusively on bioclimatic variables, evaluating their viability under the projected future climate conditions under the SSP2-4.5 and SSP5-8.5 scenarios.

Correlations among environmental variables could result in misinterpretation, overfitting, and increased MaxEnt complexity, potentially simulating an unrealistic variety of suitable habitats for species [53,54]. To effectively address multicollinearity among the predictor variables, we integrated values derived from coupling environmental variables with occurrence data [54]. By employing Pearson’s correlation coefficient and calculating Variance Inflation Factors (VIF) using the “usdm” package in R [55,56], the appropriate variables were chosen for each species with a correlation coefficient <  0.8, a common threshold in SDMs [57], and a VIF <  10. Finally, the five variables were determined for each landscape tree (Table 1).

**Table 1:** Bioclimatic variables used for MaxEnt modeling.

Variable	Description	Unit	Species
**Bio03**	Isothermality (Bio02/Bio07) (*100)	**/**	***S, B, T***
**Bio04**	**Temperature Seasonality (standard deviation** * **100)**	**/**	***S***
**Bio06**	**Min Temperature of the Warmest Monty**	**°C**	***B***
**Bio08**	**Mean Temperature of the Wettest Quarter**	**°C**	***S, B, T***
**Bio09**	**Mean Temperature of the Driest Quarter**	**°C**	***T***
**Bio13**	**Precipitation of the Wettest Month**	**mm**	***S, B, T***
**Bio14**	**Precipitation of the Driest Month**	**mm**	***S, T***
**Bio15**	**Precipitation Seasonality (coefficient of variation)**	**/**	***B***

The ‘Species’ column represents which species each variable was used for: “S” for *Stewartia koreana* Nakai ex Rehder (Theaceae); “B” for *Betula ermanii* Cham. (Betulaceae); “T” for *Taxus cuspidate* Siebold & Zucc. (Taxaceae).

### 2.3. Model optimization

Default settings are generally employed when forecasting the potential spatial range of species with MaxEnt [56]. However, models constructed using these default parameters often become overly complex, potentially leading to overfitting [58–60]. Two primary adjustable parameters crucial to the functionality of MaxEnt are the Regularization Multiplier (RM) and Feature Classes (FC). The RM introduces additional restrictions to the model, effectively acting as a penalization mechanism, while FCs apply mathematical transformations to the covariates within the model, enabling the representation of intricate relationships [61,62]. In our research, we utilized ENMeval 2.0 [25] to optimize the parameters of the MaxEnt model, selecting FC and RM values specific to each species to customize the model effectively [8,27,63,64].

First, we partitioned the occurrence data for the three species utilizing the “block” method, which divides the data into four spatial groups along latitude and longitude lines, each containing nearly equal number of records [25]. This method allowed us to segregate the data into testing and training sets.

To optimize the parameters of the MaxEnt model, we adjusted the RM within a range from 0.5–4, in increments of 0.5 to obtain eight RM values. We explored different FCs, including L, LQ, H, LQH, LQHP, and LQHPT (L = linear, Q = quadratic, H = hinge, P = product, and T = threshold), resulting in 6 FC values. Collectively, this resulted in 48 parameter configurations (8 RM ×  6 FC) [8,54,56]. We utilized ENMeval 2.0 to evaluate the 48 parameter configurations, comprehensively assessing their impact on model performance. The model’s adequacy and complexity were assessed using Akaike Information Criterion correction (AICc) [65–67]. To assess model overfitting, we considered the difference between the Area Under the Curve (AUC.diff) of the training and testing datasets, along with the 10% training omission rate (OR10) [8,27 ,54,56,59,68,69]. The optimal parameter set for model construction was determined by identifying the combination of parameters with the lowest delta AICc values.

### 2.4. MaxEnt model simulation and performance evaluation

We imported occurrence records (187 for *S. koreana*, 109 for *B. ermanii*, and 218 for *T. cuspidata*) along with the selected bioclimatic variable raster sets into MaxEnt 3.4.4 [70] to perform simulations. Specifically, we analyzed 187 distribution records and five environmental variables for *S. koreana*, 109 records and five variables for *B. ermanii*, and 218 records and five variables for *T. cuspidata* (Table 1)*.* To project future climates under the SSP2-4.5 and SSP5-8.5 scenarios, we built identical testing and training datasets to implement cross-validation and ensure consistency in the changes attributed to the SSP scenarios. This approach allowed us to maintain the same testing and training data across projections (i.e., ensuring that the SSP2-4.5 2020s and SSP5-8.5 2020s projections utilized identical sets of testing and training data). For each species, occurrence data were divided into training and testing sets at an 80:20 ratio, with a maximum of 10,000 background points. Additionally, to ensure sufficient time for the prediction results to converge, the maximum iteration value was set to 5,000 [71,72]. The data output format was set to “Logistic”, and the final results were determined by averaging the outcomes from 10 iterations.

The model’s performance was evaluated using the Area Under the receiver operating Characteristic (AUC) curve, a metric preferred for its independence from any specific thresholds [73–75]. A random probability distribution typically yields an AUC of 0.5, whereas a perfect model achieves an optimal AUC of 1.0. Any model with an AUC greater than 0.75 is considered to have potential practical utility [76].

### 2.5. Climatic sustainable suitability area

Projections were conducted at 10-year interval, from the 2020s to the 2090s, utilizing optimized MaxEnt models to assess the sustainability of climate suitability areas for *S. koreana*, *B. ermanii*, and *T. cuspidata* under the SSP2-4.5 and SSP5-8.5 climate change scenarios. Using QGIS 3.22.8 [77], the simulation results were classified with the Jenks natural breaks method into four categories of suitability: high suitability (0.6 ≤  x ≤  1), medium suitability (0.4 ≤  x <  6), low suitability (0.2 ≤  x <  0.4), and no suitability (x <  0.2), where x refers to the predicted probability of presence [22,54,78,79]. To investigate climate sustainability, the suitability maps were transformed into binary format, with high and medium suitability set to “1” and low and no suitability set to “0.” The eight binary maps for the 2020s to the 2090s were overlaid in a stacked format, creating a map with values ranging from a maximum of 8 (11111111) to a minimum of 0 (00000000). This visualization distinguishes between: (1) “Sustainable suitability”, maintaining suitability from the 2020s to the 2090s; (2) “Risk”, exhibiting suitability from the 2020s but declining thereafter; (3) “Inflow”, initially unsuitable, becoming suitable by the 2090s; (4) “Lost”, demonstrating suitability only in the 2020s, diminishing afterward; and (5) “Variable”, characterized by irregular suitability fluctuations.

## 3. Results

### 3.1. Optimal models and accuracy for *S. koreana*, *B. ermanii*, and *T. cuspidata*

**Fig 2:**
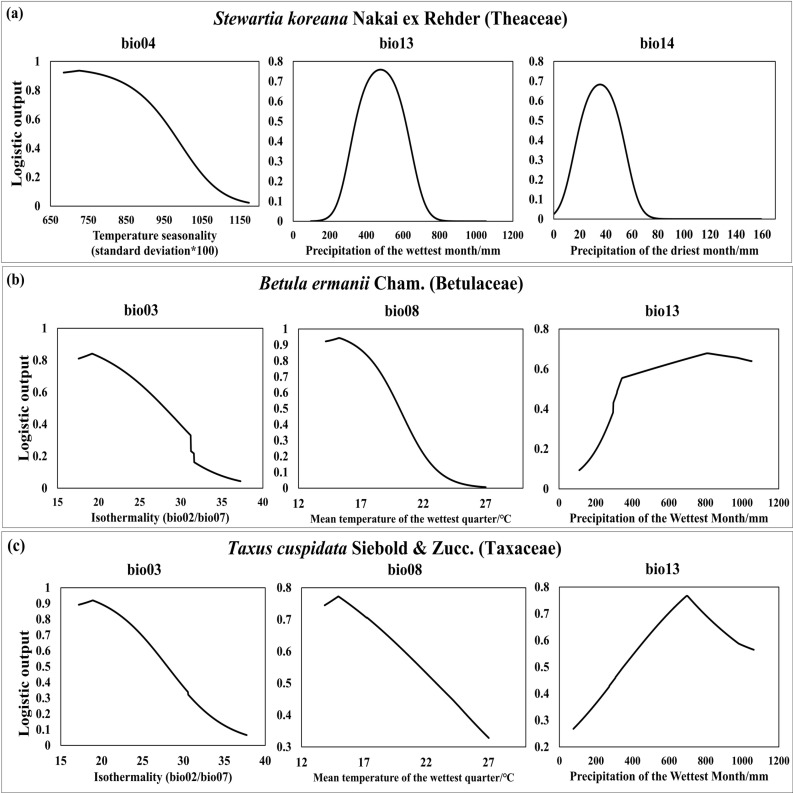
The key influential variables identified for *Stewartia koreana* Nakai ex Rehder (Theaceae), *Betula ermanii* Cham. (Betulaceae), and *Taxus cuspidata* Siebold & Zucc. (Taxaceae).

To forecast potentially suitable habitats for *S. koreana*, *B. ermanii*, and *T. cuspidata* within their distinct regions, the minimum AICc value guided the selection of the most effective feature combination. The model initially used the default parameters of RM = 1 and FC=LQHPT. Following optimization, adjustments were made to enhance accuracy: the parameters for *S. koreana* were optimized to RM = 0.5, FC=LQ; for *B. ermanii* to RM = 2.5, FC=LQHPT; and for *T. cuspidata* to RM = 2.5, FC=LQHPT, all achieving a delta.AICc = 0 (Table 2). For *S. koreana,* the AUC.diff.avg and OR10.avg values decreased by 6.24% and 35.72%, respectively, compared to the default settings. For *B. ermanii*, these values decreased by 4.07% and 19.91%, respectively, and for *T. cuspidata* by 30.18% and 27.28%, respectively (Fig 2a and 2b). The MaxEnt model was iterated 10 times after optimizing parameters. The outcomes revealed an average testing AUC of 0.875 ± 0.0271 for *S. koreana*, 0.896 ± 0.0347 for *B. ermanii*, and 0.798 ± 0.0318 for *T. cuspidata* S2 Fig, all significantly surpassing the baseline AUC of 0.5 for random classification, indicating significant predictive accuracy.

**Table 2:** Comparison of key MaxEnt values between the default settings and the optimized parameters selected for *Stewartia koreana* Nakai ex Rehder (Theaceae), *Betula ermanii* Cham. (Betulaceae)*,* and *Taxus cuspidata* Siebold & Zucc. (Taxaceae)*.*

Species	Type	RM	FC	delta.AICc	AUC.diff.avg	OR10.avg
*S. koreana*	default	1	LQHPT	117.915	0.06167	0.14894
	**optimized**	**0.5**	**LQ**	**0**	**0.05782**	**0.09574**
*B. ermanii*	default	1	LQHPT	81.529	0.04276	*0.13624*
	**optimized**	**2.5**	**LQHPT**	**0**	**0.04102**	***0.10912***
*T. cuspidata*	default	1	LQHPT	33.315	0.08097	*0.2*
	**optimized**	**2.5**	**LQHPT**	**0**	**0.05653**	***0.14545***

### 3.2. Contribution of bioclimatic variables and response curve analysis

The jackknife technique was employed to examine the influence of bioclimatic variables S3 Fig. For *S. koreana*, Temperature Seasonality (Bio04), Precipitation of the Wettest Month (Bio13), and Precipitation of the Driest Month (Bio14) emerged as the most influential among the five bioclimatic variables analyzed, contributing 84.5% of the cumulative percent contribution and 98.6% of the cumulative permutation importance S2 Table. For *B. ermanii*, the Mean Temperatures of the Wettest Quarters (Bio08), Bio13, and Isothermality (Bio03) were pivotal, accounting for a cumulative contribution of 85% and a permutation importance of 83% S2 Table. Similarly, for *T. cuspidata*, Bio03, Bio08, and Bio13 were significant, with a cumulative contribution of 89.5% and a permutation importance of 76.5% S2 Table. The jackknife test and percent contribution analysis revealed that Bio04, Bio13, and Bio14 for *S. Koreana*, and Bio03, Bio08, and Bio13 for *B. ermanii* and *T. cuspidata* had a significant impact on their future distribution based on current climate conditions and occurrence data (Fig 2).

In Fig 2a, Temperature Seasonality (Bio04) consistently shows a negative correlation with *S. koreana*. In contrast, Precipitation in the Wettest Month (Bio13) demonstrates a positive correlation up to a threshold of 477.17 mm, beyond which it shifts to a negative correlation. Similarly, Precipitation in the Driest Month (Bio14) shows a positive correlation up to 35.42 mm, after which it becomes negative. Moreover, both Bio13 and Bio14 exhibit a trend of sharp convergence toward zero after surpassing their respective thresholds. This suggests that although precipitation has a beneficial impact on *S. koreana*, extreme increases can lead to adverse consequences.

**Fig 3:**
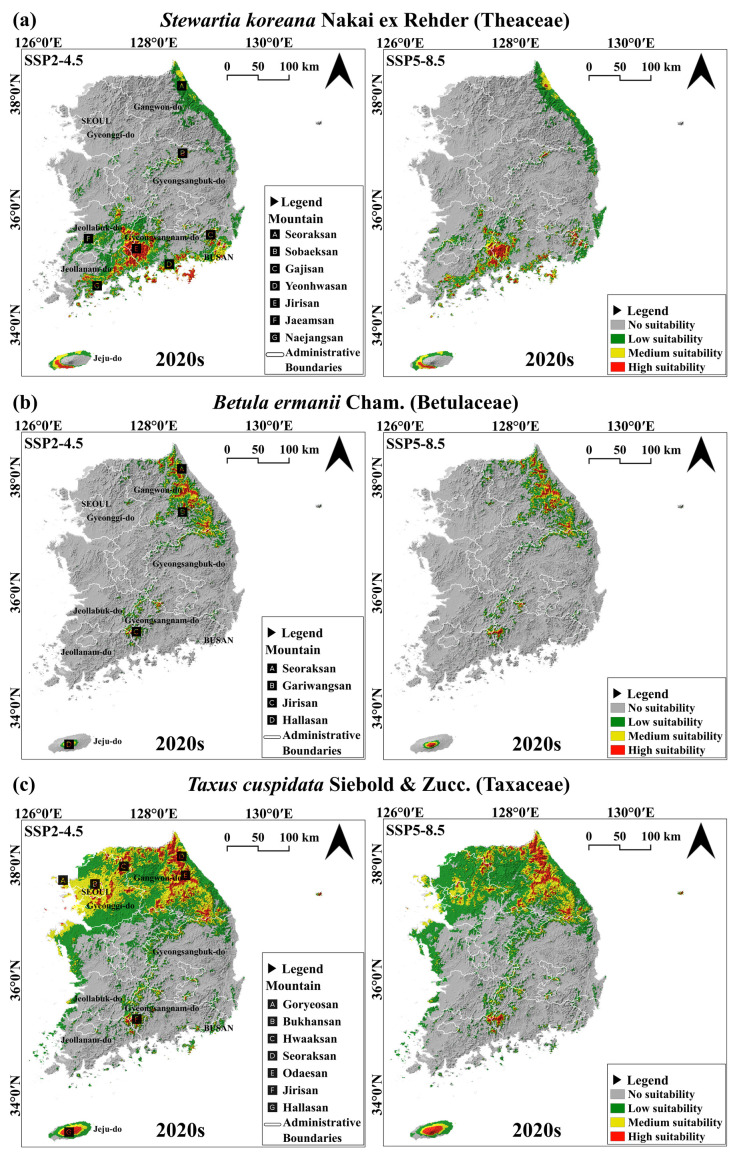
Projections of climate suitability for three species in the 2020s.

For the analysis of climatic suitability, models were constructed using bioclimatic variables spanning the same period as the collected species data (2000–2019), and projections were made for the 2020s (2000–2030). Subsequently, probability distributions were reclassified into discrete categories, namely high suitability (0.6 ≤  x ≤  1), medium suitability (0.4 ≤  x <  0.6), low suitability (0.2 ≤  x <  0.4), and no suitability (x <  0.2). For *B. ermanii*, Isothermality (Bio02/Bio07) (Bio03) and the Mean Temperature of the Wettest Quarter (Bio08) show a negative correlation, whereas Precipitation of the Wettest Month (Bio13) shows a positive correlation (Fig 3b). Notably, an increase in summer precipitation had a significantly positive impact. This pattern was similarly observed for *T. cuspidata* (Fig 3c), with both species experiencing adverse effects as temperatures increased. The threshold values at which *B. ermanii* and *T. cuspidata* begin to experience adverse effects on their potential climate suitability are as follows: for *B. ermanii*, Bio03 >  19.22, Bio08 >  15.26 °C, Bio13 >  809.16 mm; for *T. cuspidata*, Bio03 >  18.92, Bio08 >  14.96 °C, Bio13 >  697.91 mm.

### 3.3. 2020s potential climate suitability areas for *S. koreana*, *B. ermanii*, and *T. cuspidata* in South Korea

To predict the future climate suitability of landscape trees, occurrence data for *S. koreana*, *B. ermanii*, and *T. cuspidata* from 2000–2019 were used as dependent variables. Independent variables were derived from climatic data corresponding to the same period as the occurrence data collection. Subsequently, optimized parameters were applied to MaxEnt using these input data to project suitability from the 2020s to the 2090s S3–S8 Figs. Additionally, the current reference period was set as the 2020s, incorporating both historical climate data (2000–2019) and near-future projected climate data (2021–2030). As a result, the 2020s also exhibit differences according to the scenarios (Fig 3).

Using the optimal parameters, the MaxEnt model prediction results were divided into four categories and visualized using QGIS, as shown in Fig 3. The red areas indicate high suitability, the yellow areas denote medium suitability, the green areas represent low suitability, and the gray areas indicate unsuitable regions.

*S. koreana* primarily shows extensive climate suitability areas centered around the Jirisan Mountain in the southern region, followed by the Sobaeksan Mountain in the central highlands and the Seoraksan Mountain in the northern region (Fig 3a). *B. ermanii* predominantly had extensive climate suitability areas along the Seoraksan and Gariwangsan Mountains in the northern region, with suitable climate areas appearing near the Jirisan Mountain in the southern region and around the Hallasan Mountain in the Jeju-do Province, an island to the south (Fig 3b). *T. cuspidata* shows a geographic distribution of high climate suitability areas similar to those of *B. ermanii* but covering a more extensive area. Notably, a wide range of suitable climate areas were also observed in the northwestern region, including the Goryeosan, Bukhansan, and Hwaaksan Mountains (Fig 3c). The predicted climate suitability for the 2020s based on the SSP2-4.5 and SSP5-8.5 scenarios revealed that the geographical locations of “Medium suitability” and “High suitability” do not exhibit significant differences, although variations in the distribution area were observed. For *S. koreana*, under the SSP2-4.5 scenario shows wider climate suitability areas were observed near the Jirisan, Yeonhwasan, and Gajisan Mountains relative to the SSP5-8.5 scenario. For *T. cuspidata*, the SSP2-4.5 scenario showed a wide range of climate suitability areas in the northwestern region, including the Goryeosan, Bukhansan, and Hwaaksan Mountains; however, under the SSP5-8.5 scenario, many of these areas were classified as “Low suitability”. Overall, the SSP5-8.5 scenario tended to reduce climate suitability areas compared to the SS245 scenario.

### 3.4. Comparison of suitability area changes over time for *S. koreana, B. ermanii,* and *T. cuspidata* based on SSP climate change scenarios

We predicted the potential climate suitability areas from the 2020s to the 2090s based on the SSP2-4.5 and SSP5-8.5 scenarios. Suitability was classified using Jenks’ natural breaks method, with areas of high and medium suitability considered suitable for human management.

**Fig 4:**
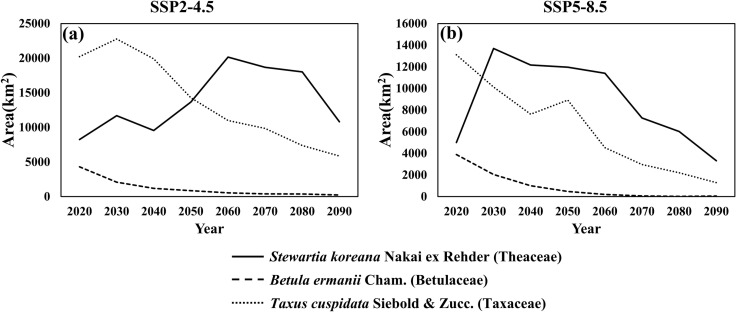
Changes in the available climate suitability areas for *Stewartia koreana* Nakai ex Rehder (Theaceae), *Betula ermanii* Cham. (Betulaceae), and *Taxus cuspidata* Siebold & Zucc. (Taxaceae).

As shown in Fig 4a, under the SSP2-4.5 scenario, the suitable habitat for *S. koreana* expanded significantly, increasing by 144% from 8,234.03 km^2^ in the 2020s to 20,219.05 km^2^ by the 2060s. This expansion occurred mainly in the southern regions, including island territories and along the eastern coastline. However, a subsequent decline of 46.4% was observed toward the 2090s, culminating in a climate suitability area of 10,804.17 km^2^. Despite this reduction, there was still an overall growth of 31.2% compared to the 2020s, attributed to the spread of suitable areas along the northern coastlines. In contrast, *B. ermanii* exhibited a consistent decline in suitable areas, with a 94.9% decrease from 4,298.5 km^2^ in the 2020s to just 218.41 km^2^ by the 2090s, indicating a significant loss in lower elevation regions. Meanwhile, *T. cuspidata* experienced a slight initial increase of 12.6% in the 2030s from its original 20,219.05 km^2^, only to undergo a sharp decline of 71.01% down to 5,861.36 km^2^ by the 2090s, reflecting a significant contraction in suitable habitats within the northern areas over the projected period.

As shown in Fig 4b, under the SSP5-8.5 scenario, *S. koreana* exhibited a broader suitable area in the 2030s and 2040s compared to the SSP2-4.5 scenario, but showed a sharp decline after the 2060s, resulting in a 33.6% decrease compared to the 2020s. In stark contrast to the SSP2-4.5 scenario, *B. ermanii* showed a decrease of 98.9%, and *T. cuspidata* showed a decrease of 90.1% by the 2090s. Notably, *B. ermanii* showed a tendency toward almost complete areas loss by the 2090s, demonstrating a more extreme trend relative to the SSP2-4.5 scenario. In summary, under SSP2-4.5, both *B. ermanii* and *T. cuspidata* showed a continuous decrease in their suitability areas, whereas *S. koreana* initially exhibited an increasing trend, which subsequently declined after peaking in the 2060s. Conversely, under SSP5-8.5, *S. koreana* also exhibited a continuous declining trend, similar to *B. ermanii* and *T. cuspidata*.

**Fig 5:**
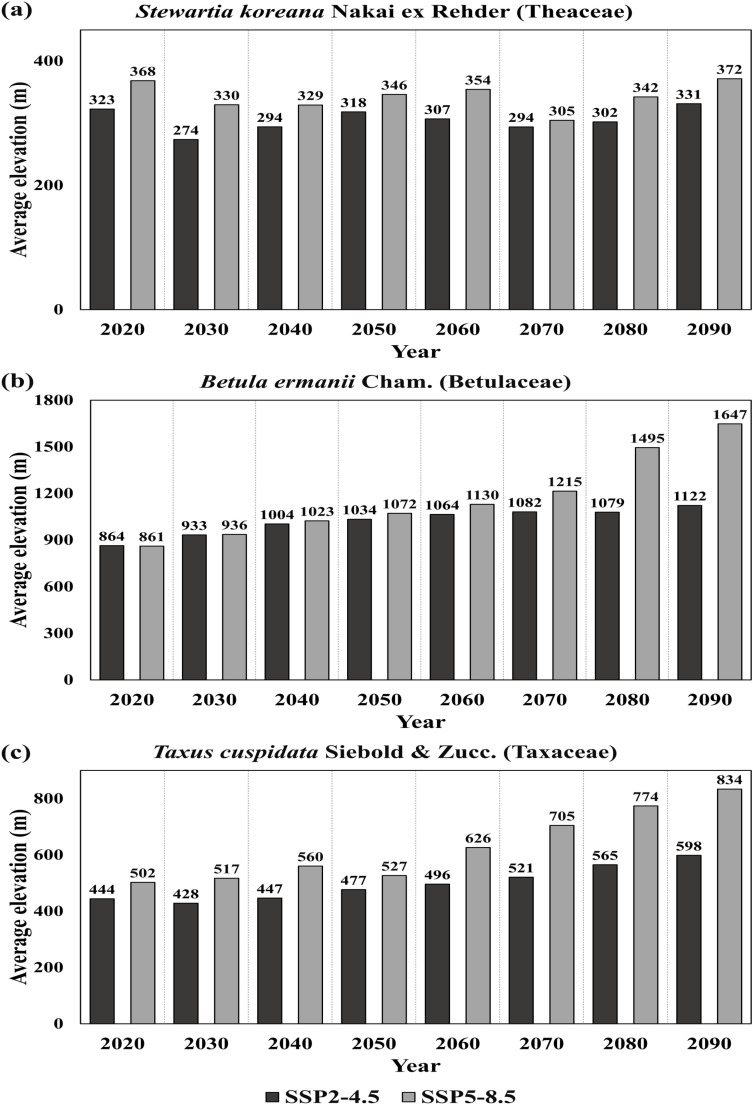
Changes in the average elevation of available climate suitability areas for *Stewartia koreana* Nakai ex Rehder (Theaceae), *Betula ermanii* Cham. (Betulaceae), and *Taxus cuspidata* Siebold & Zucc. (Taxaceae).

In order to investigate the impact of different climate change trajectories on elevation shifts, the time trend of the average elevation in climate suitability areas was compared between climate scenarios for the three species (Fig 5). The average elevation of SSP5-8.5 scenario was consistently higher than that in the SSP2-4.5 scenario across all time periods and for all species. The average elevation showed an increasing trend as the area decreased. For *S. koreana*, however, a distinct trend of elevation shift over time was not observed, with elevation decline between 2060 and 2070 under the SSP5-8.5 scenario while climate suitability areas decrease (Fig 5a). Additionally, there was no increase of elevation for *S. koreana* from 2020 to 2090. As seen in S6 and S7 Figs, “Medium suitability” and “High suitability” areas for *S. koreana* under both scenarios shrank in size but largely remained within the 2020s distribution, resulting in minimal changes in average elevation. For both *B. ermanii* and *T. cuspidata*, there was increasing trend in average elevation over time and the difference between SSP2-4.5 and SSP5-8.5 scenarios grew larger as time progressed (Fig 5b and 5c). In contrast, while the climate suitability areas of *T. cuspidata* shrink gradually (Fig 4a), little change in average elevation was observed from 2070 to 2090 (Fig 5c). However, only the climate suitability areas at higher elevation persisted under the SSP5-8.5 scenario, leading to an increase in average elevation. As show in S5 and S8 Figs, Unlike the other two species, *B. ermanii* showed very limited climate suitability areas concentrated in high elevation regions under both scenarios. In particular, Under the SSP5-8.5 scenario, most of its climate suitability areas were nearly lost. Instead of focusing on *B. ermanii*’s potential use as future landscape tree, efforts should prioritize the conservation and enhanced management of its existing populations.

### 3.5. Sustainable future climate suitability areas for *S. koreana, B. ermanii,* and *T. cuspidata* based on SSP climate change scenarios

**Fig 6:**
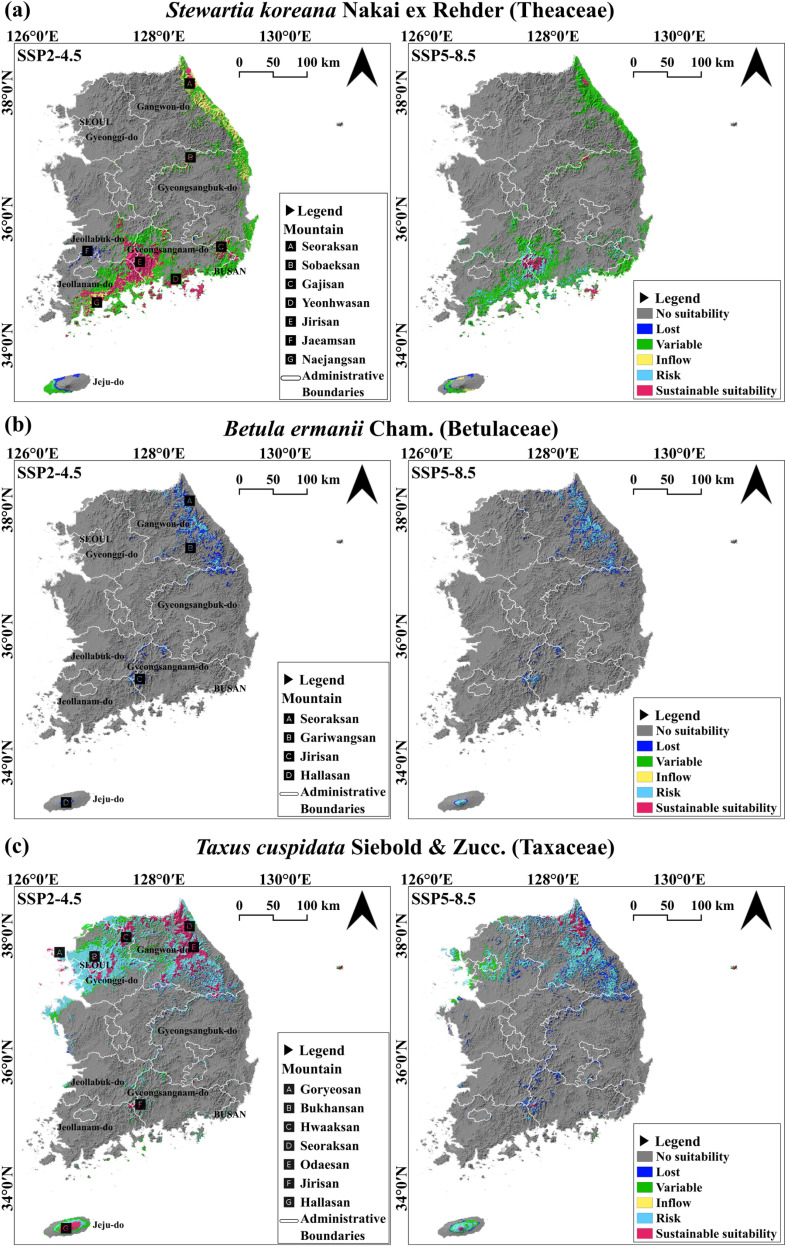
Classification of suitability areas from the 2020s–2090s based on the SSP2-4.5 and SSP5-8.5 scenarios. “No suitability”: regions that become unsuitable from 2020–2090s. “Lost”: areas that are suitable in the 2020s but lose suitability thereafter. “Risk”: areas that lose suitability after a specific time point. “Inflow”: regions that become suitable after a specific time point. “Sustainable suitability”: areas that remain suitable from the 2020s–2090s. “Variable”: regions with fluctuating suitability, including periods of both influx and decline.

We overlaid suitability areas from the 2020s–2090s and reclassified them into different categories. “Lost” represents regions that were suitable in the 2020s but ceased to be suitable thereafter. “Risk” denotes areas that remained consistently suitable from the present up to a certain point, after which they became unsuitable. “Inflow” refers to regions that are not currently suitable but become suitable after a specific time point and remain suitable thereafter. “Sustainable suitability” encompasses areas that consistently remained suitable from the present to 2090s. “Variable” represents regions with fluctuating suitability, including areas experiencing both influx and decline. Following this reclassification, we conducted a comparative analysis of these areas (Fig 6).

**Fig 7:**
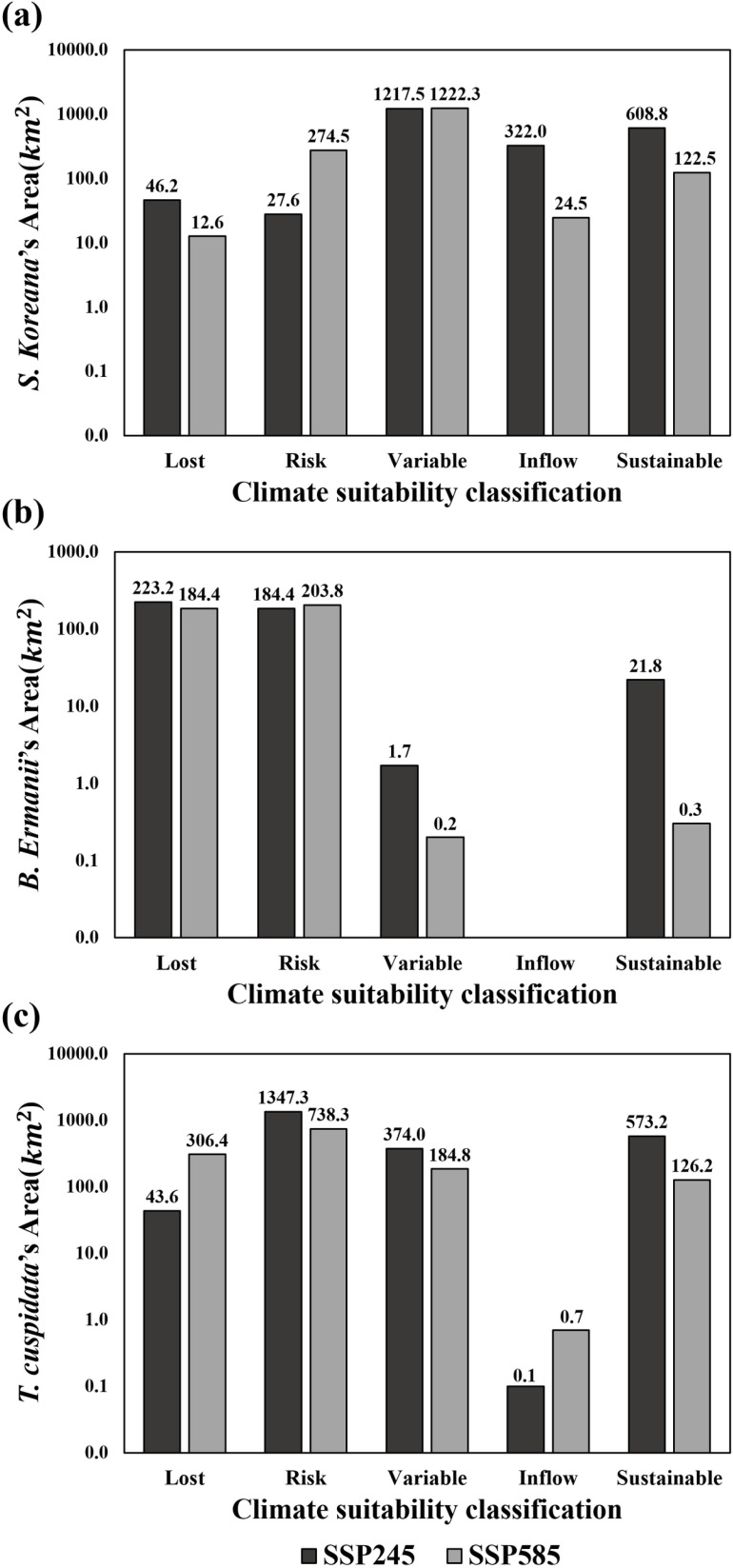
Comparison of changes in suitability areas for *Stewartia koreana* Nakai ex Rehder (Theaceae), *Betula ermanii* Cham. (Betulaceae), and *Taxus cuspidata* Siebold & Zucc. (Taxaceae) across the SSP2-4.5 and SSP5-8.5 scenarios.

Under the SSP2-4.5 scenario, “Sustainable suitability” for *S. koreana* was identified in the southern regions, including the Jaeamsan, Jirisan, Yeonhwasan, and Gajisan Mountains, as well as in the central region around the Sobaeksan Mountain and the northeastern region around the Seoraksan Mountain (Fig 6a). In contrast, under the SSP5-8.5 scenario, “Sustainable suitability” was found in the Jirisan, Gajisan, Sobaeksan, and Seoraksan Mountains (Fig 6a). Notably, there was a 66.5% difference in the area of “Sustainable suitability” between the two scenarios (Fig 7a). This difference is attributed to the transition of suitability in the Jaeamsan, Yeonhwasan, and Gajisan Mountains in the southern region from “Sustainable suitability” to “Risk” and “Variable” categories. Additionally, under the SSP2-4.5, the area classified as “Lost”, which showed suitability in the 2020s but lost it thereafter, was 57.1% higher compared to the SSP5-8.5 scenario (Fig 7a), with most of this area located near the Naejangsan Mountain (Fig 6a). This region transitioned to “Risk” and “Variable” categories under the SSP5-8.5 scenario (Fig 6a). The “Inflow” areas, which did not show suitability initially but became suitable and remained so thereafter, showed an 85.9% larger difference between the scenarios (Fig 7a), with “Inflow” along the east coast up to the northern region under the SSP2-4.5 (Fig 6a). In SSP5-8.5, this region mostly transitioned to the “Variable” category (Fig 6a).

For *B. ermanii*, under the SSP2-4.5 (Fig 6b), “Sustainable suitability” was identified in a few high-elevation over 1200 m, such as the Jirisan and Hallasan Mountains in the south and the Gariwangsan and Seoraksan Mountains in the north. In the SSP5-8.5, “Sustainable suitability” was further narrowed to the Hallasan Mountain (Fig 6b). No “Inflow” areas were identified for *B. ermanii*, and both scenarios predominantly showed “Risk” and “Lost” suitability (Fig 6b). The total suitable area was 5.2% (Fig 7b), showing no significant difference between the two scenarios, suggesting that *B. ermanii’*s future climate suitability in South Korea is expected to continuously decrease due to climate change.

For *T. cuspidata*, under the SSP2-4.5 scenario (Fig 6c), “Sustainable suitability” was found in the northwest regions of the Goryeosan, Bukhansan, and Hwaaksan Mountains, the northeastern regions of the Seoraksan and Odaesan Mountains, and the southern regions of the Jirisan and Hallasan Mountains. Under the SSP5-8.5 scenario, “Sustainable suitability” decreased by 63.9% (Fig 7c), with differences arising as the Goryeosan, Bukhansan, Hwaaksan Mountains in the northwest, and areas around Jirisan and Hallasan Mountains in the south transitioned to “Risk” or “Variable” categories or became unsuitable (Fig 6c). The “Inflow” areas for *T. cuspidata* are not expected to be significant in either scenario. Moreover, there was a substantial difference in the “Lost” and “Risk” areas between the two scenarios. Under the SSP2-4.5 scenario, areas showing suitability in the 2020s are expected to maintain suitability for a relatively short-term future, whereas under the SSP5-8.5, areas losing suitability increased by 75.1% compared to SSP2-4.5 (Fig 7c). Additionally, under the SSP2-4.5 scenario, there were extensive “Risk” areas in the northwest, which transitioned to “No suitability” under SSP5-8.5. The “Sustainable suitability” areas in SSP2-4.5 also shifted to “Risk” in the long-term future, highlighting significant differences between the scenarios.

## 4. Discussion

### 4.1. Optimizing MaxEnt model performance

In this study, we utilized MaxEnt to predict potential climate suitability areas for *S. koreana*, *B. ermanii*, and *T. cuspidata* in South Korea. MaxEnt is particularly adept at deriving valuable insights from minimal sample sizes and small datasets [80]. This capability includes generating species-specific response curves and quantitatively evaluating environmental variables that define suitable habitats [8,20,81]. To further minimize errors, we utilized ENMeval, which systematically optimizes the MaxEnt model’s complexity through the interaction of various parameters [64,82–84].

The RM was set to eight values ranging from 0.5–4, and the FCs adopted six feature combinations: L, LQ, H, LQH, LQHP, and LQHPT, resulting in the examination of 48 parameter combinations. In this study, the MaxEnt parameters identified using ENMeval were as follows: for *S. koreana*, RM = 0 and FC=LQ; for *B. ermanii*, RM = 2.5 and FC=LQHPT; and for *T. cuspidata*, RM = 2.5 and FC=LQHPT. Notably, the delta.AICc value was 0 at this stage. Model parameters were established by considering species characteristics and data structure. Previous reports suggest that incorporating segmented features (H) into FC settings can significantly enhance the model’s transferability [85]. Additionally, the RM value is typically higher than MaxEnt’s default setting of 1, with models using RM values in the range of 2–4 demonstrating the best performance [58,86,87]. In this study, for *B. ermanii* and *T. cuspidata*, the RM values fell within the range of 2–4, and their FC values included segmented features (H). These similarities are justified given that both species exhibit broad distribution and have similar occurrence locations. However, the skewed occurrence of *S. koreana* toward the south likely influenced its RM and FC values.

### 4.2. Influences of critical environmental variables on the distributions of *S. koreana*, *B. ermanii*, and *T. cuspidata*

Based on the MaxEnt model’s contribution rates, permutation importance, and jackknife tests, the significant climatic factors affecting the distribution of *S. koreana* were identified as Temperature Seasonality (Bio04), Precipitation of the Wettest Month (Bio13), and Precipitation of the Driest Month (Bio14). For *B. ermanii* and *T. cuspidata*, the Mean Temperature of the Wettest Quarter (Bio08), Isothermality (Bio02/Bio07) (Bio03), and Precipitation of the Wettest Month (Bio13) emerged as the main climatic factors influencing their distribution. In summary, the key climatic variables used to predict the climate suitability areas for the three species were Bio03, Bio04, Bio08, Bio13, and Bio14. According to O’Donnel and Ignizio [88], Bio03 refers to the difference between the diurnal and annual temperature ranges. A value close to 100 indicates that the diurnal temperature range is not significantly different from the annual temperature range, suggesting an environment in which temperatures remain relatively constant. Bio04 represents the variation in temperature between seasons, with higher values indicating increased temperature fluctuations throughout the year and a notable impact of seasonal changes. Bio08 refers to the average temperature during the wettest quarter, which in South Korea typically includes the summer months (June, July, and August). Bio13 represents the amount of precipitation during the same quarter as Bio08. Finally, Bio14 indicates the average precipitation during the driest quarter, which in South Korea is usually the winter months (December, January, and February).

*S. koreana* appears to be less suited to climates with significant seasonal temperature variations, particularly when summer and winter precipitation exceed 477.17 mm and 35.42 mm, respectively. In contrast, *B. ermanii* and *T. cuspidata* showed a decrease in suitability as the environment became more temperature-stable and were similarly unsuitable for hot and humid conditions characterized by high temperatures and substantial rainfall during the summer. The regions where these species occur in South Korea are particularly vulnerable to the effects of climate change [6,89]. Moreover, South Korea is also susceptible to the climatic influences of neighboring China. Recently, Northeast China’s climate has experienced warming and drying trends, posing potential risks of intensified heat waves and drought conditions in South Korea as well [6,90].

According to Takahashi et al. [91], *B. ermanii* may encounter growth challenges due to high temperatures and significant summer rainfall. Similarly, Kong [40] reported that *T. cuspidata* experiences problems with growth and fruiting as summer temperatures rise. These characteristics align with the results of this study, reflecting the environmental sensitivity of these species. While *S. koreana* primarily thrives in the warmer environments of South Korea, it exhibits resilience to temperatures as low as − 27.6 °C. Furthermore, as South Korea’s climate gradually shifts toward subtropical regions, it is anticipated that the *S. koreana*’s suitability range will expand. However, the results of this study indicate that the area may expand to a certain extent before decreasing, possibly influenced by precipitation data bias in the collected occurrence data. Nonetheless, given the scarcity of research on *S. koreana*’s ecology, the model results based on the input data appear satisfactory.

### 4.3. Climate suitability area change

Like native and vulnerable tree species, landscape trees are also affected by climate change, prompting the use of SDMs and GIS mapping to identify tree species suitable for urban environments [33]. This study aimed to identify regions showing consistent suitability for different landscape tree species in response to climatic changes. From the 2020s–2090s, areas showing continuous climate suitability for the three species studied significantly decreased in both scenarios (SSP2-4.5 and SSP5-8.5) compared to the 2020s. SSP2-4.5 is characterized by intermediate greenhouse gas emissions, with mitigated climate change and moderate socioeconomic development, while SSP5-8.5 is characterized by very high greenhouse gas emissions, increased use of fossil fuels, and uncontrolled development [1]. Notably, both scenarios assume that climate change has progressed beyond the present. Additionally, it has been reported that the average temperature increase in South Korea has been significantly higher than the global average [89]. Consequently, it can be inferred that southern species may migrate northward, while northern species may shift their suitable habitats beyond South Korea to more northern areas. Currently, in South Korea, *S. koreana* is known as a southern species, while *B. ermanii* and *T. cuspidata* are known as northern species [39,92,93]. According to Figs 4 and 6, under the SSP2-4.5 scenario, *S. koreana* shows new suitable areas emerging along the northeastern coastline as “Inflow” areas expand compared to the 2020s, with extensive “Sustainable suitability” areas observed in the southern regions. In contrast, *B. ermanii* shows a decline in long-term suitability, with previously suitable areas in the 2020s transitioning to “Risk” and “Lost” categories, except for some high elevation regions. This suggests that with a global temperature increase of >  3 °C due to carbon emissions, *B. ermanii* is expected to lose its climate suitability in South Korea. Although *T. cuspidata* is a northern species, it maintains broad “Sustainable suitability” areas around the Hallasan, Jirisan, and Seoraksan Mountains. However, under the SSP5-8.5 scenario, the “Sustainable suitability” for *S. koreana* in the southern regions diminishes and transitions to “Risk” or “Variable” areas, with the “Inflow” regions in the north mostly converting to “Variable”. *B. ermanii*’s “Sustainable suitability” areas shift to “Risk”, and *T. cuspidata* also sees a reduction in “Sustainable suitability”, with “Risk” areas changing to “No suitability” or shrinking to only high elevation regions in the south, maintaining suitability only in parts of the Seoraksan Mountain in the north. These results indicate that suitable habitats for these species are likely to shift northward within South Korea as temperatures rise. Furthermore, with a future temperature increase of >  5.2 °C due to carbon emissions, even *S. koreana*, the southern species, shows a tendency for reduced “Sustainable suitability”. Therefore, the continuous rise in temperature in South Korea is expected to negatively impact the future climate suitability of *S. koreana*, *B. ermanii*, and *T. cuspidata*.

The results indicate that the climate suitability of landscape trees is likely to shifts due to climate change. By assessing climate suitability in advance, placement and management strategies for target species can be developed for each category, represented as “Sustainable suitability”, “Risk”, “Inflow”, “Lost”, and “Variable”. The “Lost” and “Risk” categories are predominantly observed in *B. ermanii* and *T. cuspidata*, indicating areas that are currently suitable but are at high risk of losing suitability in the future or have already lost it entirely. Consequently, new plantings of the landscape tree, representing similar pattern with *B. ermanii* and *T. cuspidata*, should be carried out carefully, while existing plantings will require intensive management. Additionally, if species with “Lost” and “Risk” areas significantly larger than other categories are identified, these species may become unsuitable for use as landscape trees in the future, resulting in the need for alternatives. Areas classified as “Inflow”, observed in *S. koreana*, are predicted to show increased suitability in the future, making them potential sites for new plantings or trial plantings. Areas classified as “Variable”, where suitability is unstable, require strategies to enhance elasticity to climate change, such as adopting mixed-species planting to mitigate risks.

### 4.4. Limitations

The primary limitation of this study was the lack of prior research on the ecological characteristics and climatic environments of the target species, making it difficult to confirm whether ecological traits were accurately reflected. Additionally, relying solely on occurrence data of native species suggests that the predicted potential suitable areas might differ from the actual suitable areas [94]. Future research incorporating data from landscape nurseries and avenue trees, which are under human management, could produce results that more closely align with the study’s objectives. The second limitation is the inability to clearly interpret the “Variable” regions from overlapping maps, and consequently, to present definitive results. Overlaying maps from the 2020s–2090s may reveal areas that were not suitable in the 2020s and 2030s but became suitable from the 2040s onwards and maintaining that suitability until the 2090s. This includes irregular results that are difficult to interpret due to the overlapping nature of the maps. Overall, this indicates that, depending on the intended use, establishing clear criteria and categorization is necessary.

## 5. Conclusions

This study utilized a MaxEnt model with species-specific optimized parameters to predict the transition from current to future climate conditions for *S. koreana*, *B. ermanii*, and *T. cuspidata*. A subsequent overlay analysis from the 2020s–2090s allowed for the examination of sustainable suitability areas. Under the SSP2-4.5 scenario, the climate suitability areas for the 2020s were 8234.03 km^2^ for *S. koreana*, 4298.53 km^2^ for *B. ermanii*, and 20219.05 km^2^ for *T. cuspidata*. However, when overlapped, the climate suitability areas demonstrating sustainable suitability by the 2090s were 608.8 km^2^, 21.8 km^2^, and 573.2 km^2^, respectively. Under the SSP5-8.5 scenario, the 2020s climate suitability areas were smaller, with *S. koreana* at 4994.67 km^2^, *B. ermanii* at 3884.6 km^2^ and *T. cuspidata* at 13109.20 km^2^. Notably, the sustainable suitability areas by the 2090s drastically decreased or disappeared, becoming 122.5 km^2^ for *S. koreana*, 0.3 km^2^ for *B. ermanii*, and 126.2 km^2^ for *T. cuspidata*. Upon examining their responses to key climatic factors, *S. koreana* showed a decrease in climate suitability with greater seasonal temperature differences, particularly when winters become very cold, and summers become very hot. Conversely, both *B. ermanii* and *T. cuspidata* exhibited reduced suitability as hot and humid summer conditions intensified. These findings highlight the species’ distinct climatic preferences, with *S. koreana* being sensitive to extreme seasonal variations, and both *B. ermanii* and *T. cuspidata* being adversely affected by increased warmth and humidity during the summer months. By confirming the changes in climate suitability due to climate change impacts, we identified a trend of decreasing sustainable suitable areas as carbon emissions scenario worsen. This underscores the necessity of considering the effects of climate change when selecting landscape trees and highlights the need for research to support rational decision-making. Additionally, this analysis can serve as a foundation for utilizing SDMs to address these climate change-associated challenges effectively.

## Supporting information

S1 Fig Selection of southern tree species for climate suitability analysis: Current distribution trends based on National Ecosystem Survey. The current distribution patterns of selected southern trees identified as climate-sensitive biologocal indicator species are based on the 2nd to 5th National Ecosystem Surveys (1997-2019). Among the 5 southern species analyzed, *Daphniphyllum macropodum* Miq. (Daphniphyllaceae), *Melia azedarach* L. (Meliaceae), *Neolitsea sericea* (Blume) Koidz. (Lauraceae), and *Machilus thunbergii* Siebold & Zucc. (Lauraceae) were consistently observed in southern regions, with minimal variation. However, *Stewartia koreana* Nakai ex Rehder (Theaceae) exhibited a notable northward distribuiton expansion, being newly recorded in central and northern regions during the 4th and 5th surveys (2014-2019).
(TIF)

S2 Fig The receiver operating characteristic (ROC) curves and area under the curve (AUC) values for the 10 replicated runs for each species. The ROC curves illustrate the performance of the MaxEnt in predicting suitable climate areas for three species: *Stewartia koreana* Nakai ex Rehder (Theaceae) (top), *Betula ermanii* Cham. (Betulaceae) (middle), and *Taxus cuspidata* Siebold & Zucc. (Taxaceae) (bottom). The mean Area Under the Curve (AUC) values for each species are 0.875, 0.896 and 0.798, respectively, indicating the model’s predictive accuracy. The blue shaded area represents the standard deviation around the mean AUC.
(TIF)

S3 Fig Jackknife test for the bioclimatic variables for each species. The Jackknife test results show the contribution of each environmental variable to the MaxEnt model’s regularized training gain for *Stewartia koreana* Nakai ex Rehder (Theaceae) (top), *Betula ermanii* Cham. (Betulaceae) (middle), and *Taxus cuspidata* Siebold & Zucc. (Taxaceae) (bottom). The blue bars represent the model’s performance with only one variable, the green bars show the performance without given variable, and the red bars indicate the performance with all variables included.
(TIF)

S4 Fig Projected climate suitability for *Stewartia koreana* Nakai ex Rehder (Theaceae) from the 2030s to the 2090s under the SSP2-4.5 scenario. The maps for *Stewartia koreana* Nakai ex Rehder (Theaceae) illustrate the projected climate suitability areas for the target species in South Korea across seven future time periods (2030, 2040, 2050, 2060, 2070, 2080, and 2090) under the SSP2-4.5 scenario. The suitability is classified into four categories: “No suitability” (gray), “Low suitability” (green), “Medium suitability” (yellow), and “High suitability” (red).
(TIF)

S5 Fig Projected climate suitability for *Betula ermanii* Cham. (Betulaceae) from the 2030s to the 2090s under the SSP2-4.5 scenario. The maps for *Betula ermanii* Cham. (Betulaceae) illustrate the projected climate suitability areas for the target species in South Korea across seven future time periods (2030, 2040, 2050, 2060, 2070, 2080, and 2090) under the SSP2-4.5 scenario. The suitability is classified into four categories: “No suitability” (gray), “Low suitability” (green), “Medium suitability” (yellow), and “High suitability” (red).
(TIF)

S6 Fig Projected climate suitability for *Taxus cuspidate* Siebold & Zucc. (Taxaceae) from the 2030s to the 2090s under the SSP2-4.5 scenario. The maps for *Taxus cuspidata* Siebold & Zucc. (Taxaceae) illustrate the projected climate suitability areas for the target species in South Korea across seven future time periods (2030, 2040, 2050, 2060, 2070, 2080, and 2090) under the SSP2-4.5 scenario. The suitability is classified into four categories: “No suitability” (gray), “Low suitability” (green), “Medium suitability” (yellow), and “High suitability” (red).
(TIF)

S7 Fig Projected climate suitability for *Stewartia koreana* Nakai ex Rehder (Theaceae) from the 2030s to the 2090s under the SSP5-8.5 scenario. The maps for *Stewartia koreana* Nakai ex Rehder (Theaceae) illustrate the projected climate suitability areas for the target species in South Korea across seven future time periods (2030, 2040, 2050, 2060, 2070, 2080, and 2090) under the SSP5-8.5 scenario. The suitability is classified into four categories: “No suitability” (gray), “Low suitability” (green), “Medium suitability” (yellow), and “High suitability” (red).
(TIF)

S8 Fig Projected climate suitability for *Betula ermanii* Cham. (Betulaceae) from the 2030s to the 2090s under the SSP5-8.5 scenario. The maps for *Betula ermanii* Cham. (Betulaceae) illustrate the projected climate suitability areas for the target species in South Korea across seven future time periods (2030, 2040, 2050, 2060, 2070, 2080, and 2090) under the SSP5-8.5 scenario. The suitability is classified into four categories: “No suitability” (gray), “Low suitability” (green), “Medium suitability” (yellow), and “High suitability” (red).
(TIF)

S9 Fig Projected climate suitability for *Taxus cuspidate* Siebold & Zucc. (Taxaceae) from the 2030s to the 2090s under the SSP5-8.5 scenario. The maps for *Taxus cuspidata* Siebold & Zucc. (Taxaceae) illustrate the projected climate suitability areas for the target species in South Korea across seven future time periods (2030, 2040, 2050, 2060, 2070, 2080, and 2090) under the SSP5-8.5 scenario. The suitability is classified into four categories: “No suitability” (gray), “Low suitability” (green), “Medium suitability” (yellow), and “High suitability” (red).
(TIF)

S1 Table The most impactful bioclimatic variables for *Stewartia koreana* Nakai ex Rehder (Theaceae), *Betula ermanii* Cham. (Betulaceae), and *Taxus cuspidate* Siebold & Zucc. (Taxaceae). This table presents the bioclimatic variable used in the species distribution modeling for *Stewartia koreana* Nakai ex Rehder (Theaceae), *Betula ermanii* Cham. (Betulaceae), and *Taxus cuspidata* Siebold & Zucc. (Taxaceae). Each variable is listed by its code (e.g., Bio03, Bio04) along with a brief description and the unit of measurement.
(DOCX)

S2 Table Percent contribution and permutation importance of each bioclimatic variable for *Stewartia koreana* Nakai ex Rehder (Theaceae), *Betula ermanii* Cham. (Betulaceae), and *Taxus cuspidate* Siebold & Zucc. (Taxaceae). This table shows the percent contribution and permutation importance of key bioclimatic variables used in the species distribution modeling for *Stewartia koreana* Nakai ex Rehder (Theaceae), *Betula ermanii* Cham. (Betulaceae), and *Taxus cuspidata* Siebold & Zucc. (Taxaceae). Percent contribution indicates the extent to which each variable influenced the MaxEnt model’s predictions, while permutation importance reflects the dependence of the model’s accuracy on each variable.
(DOCX)
