# Supplementary figures and images for: Assessment of climate change impact on landscape tree distribution and sustainability in South Korea using MaxEnt-based modeling
Source: PLoS One. 2025 Mar 3;20(3):e0316393. doi: 10.1371/journal.pone.0316393 (PMC11875377; doi:10.1371/journal.pone.0316393)

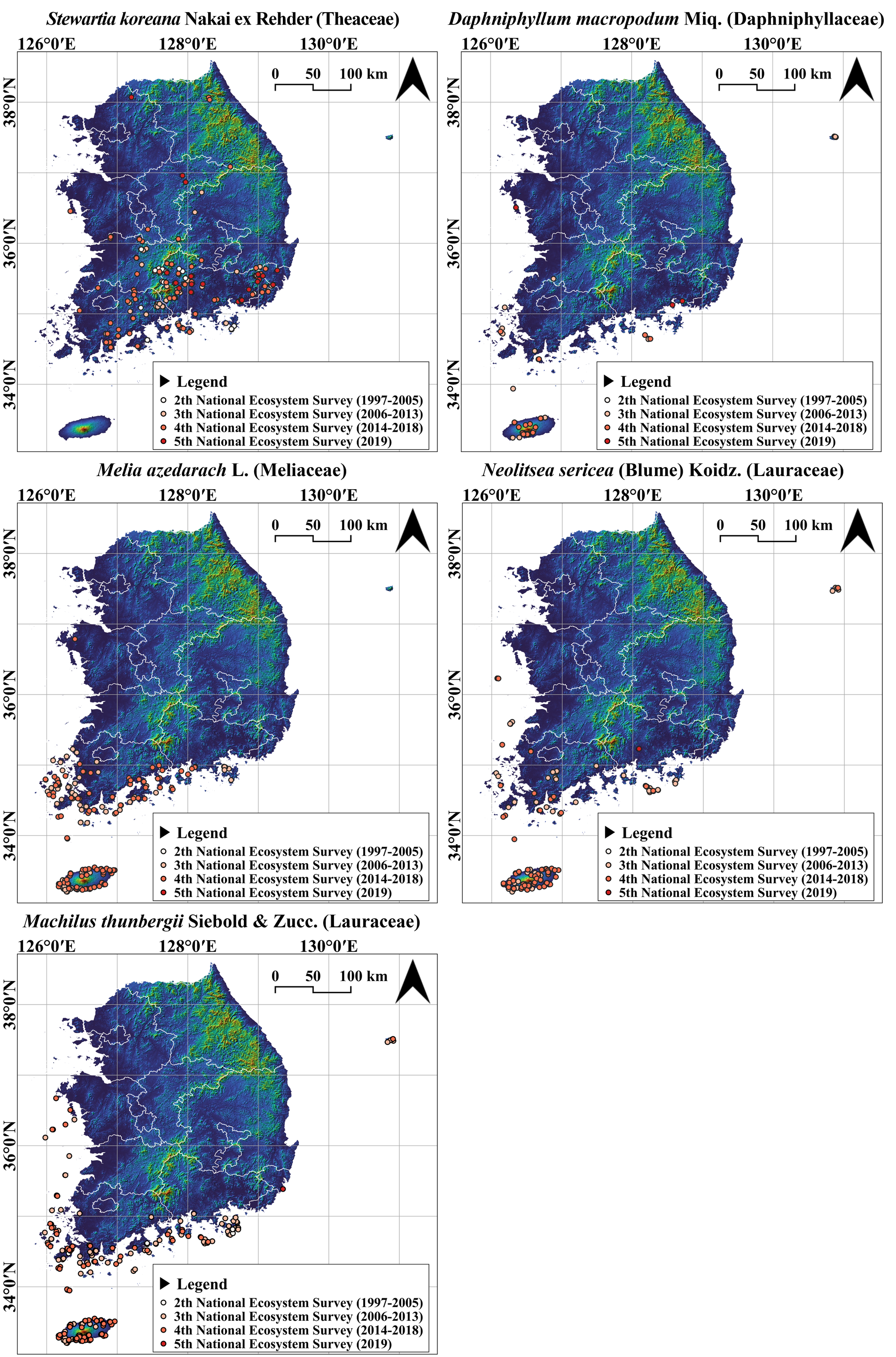

Supplement: S1 Fig — The current distribution patterns of selected southern trees identified as climate-sensitive biologocal indicator species are based on the 2nd to 5th National Ecosystem Surveys (1997-2019). Among the 5 southern species analyzed, Daphniphyllum macropodum Miq. (Daphniphyllaceae), Melia azedarach L. (Meliaceae), Neolitsea sericea (Blume) Koidz. (Lauraceae), and Machilus thunbergii Siebold & Zucc. (Lauraceae) were consistently observed in southern regions, with minimal variation. However, Stewartia koreana Nakai ex Rehder (Theaceae) exhibited a notable northward distribuiton expansion, being newly recorded in central and northern regions during the 4th and 5th surveys (2014-2019). (TIF) [file pone.0316393.s001.tif]

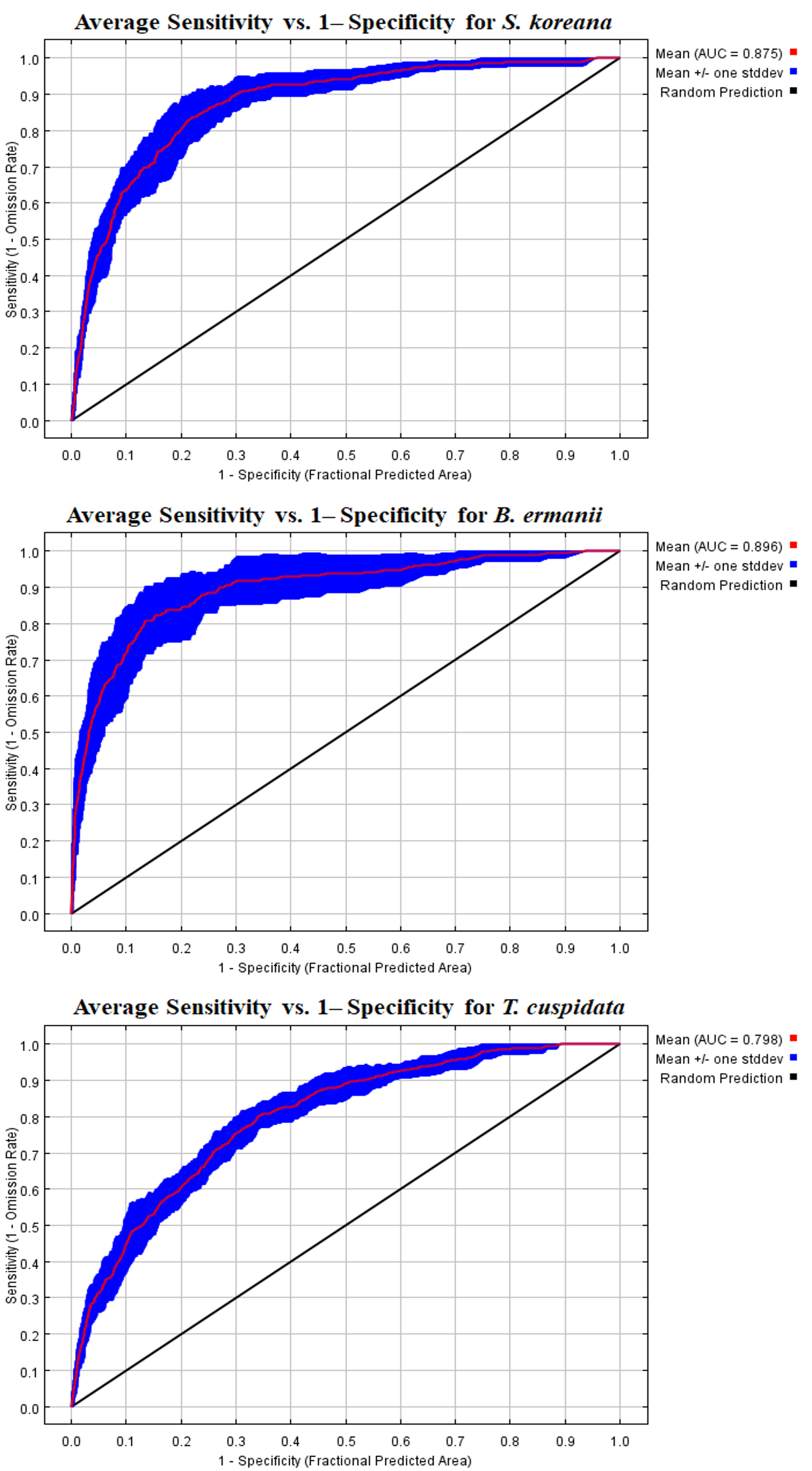

Supplement: S2 Fig — The ROC curves illustrate the performance of the MaxEnt in predicting suitable climate areas for three species: Stewartia koreana Nakai ex Rehder (Theaceae) (top), Betula ermanii Cham. (Betulaceae) (middle), and Taxus cuspidata Siebold & Zucc. (Taxaceae) (bottom). The mean Area Under the Curve (AUC) values for each species are 0.875, 0.896 and 0.798, respectively, indicating the model’s predictive accuracy. The blue shaded area represents the standard deviation around the mean AUC. (TIF) [file pone.0316393.s002.tif]

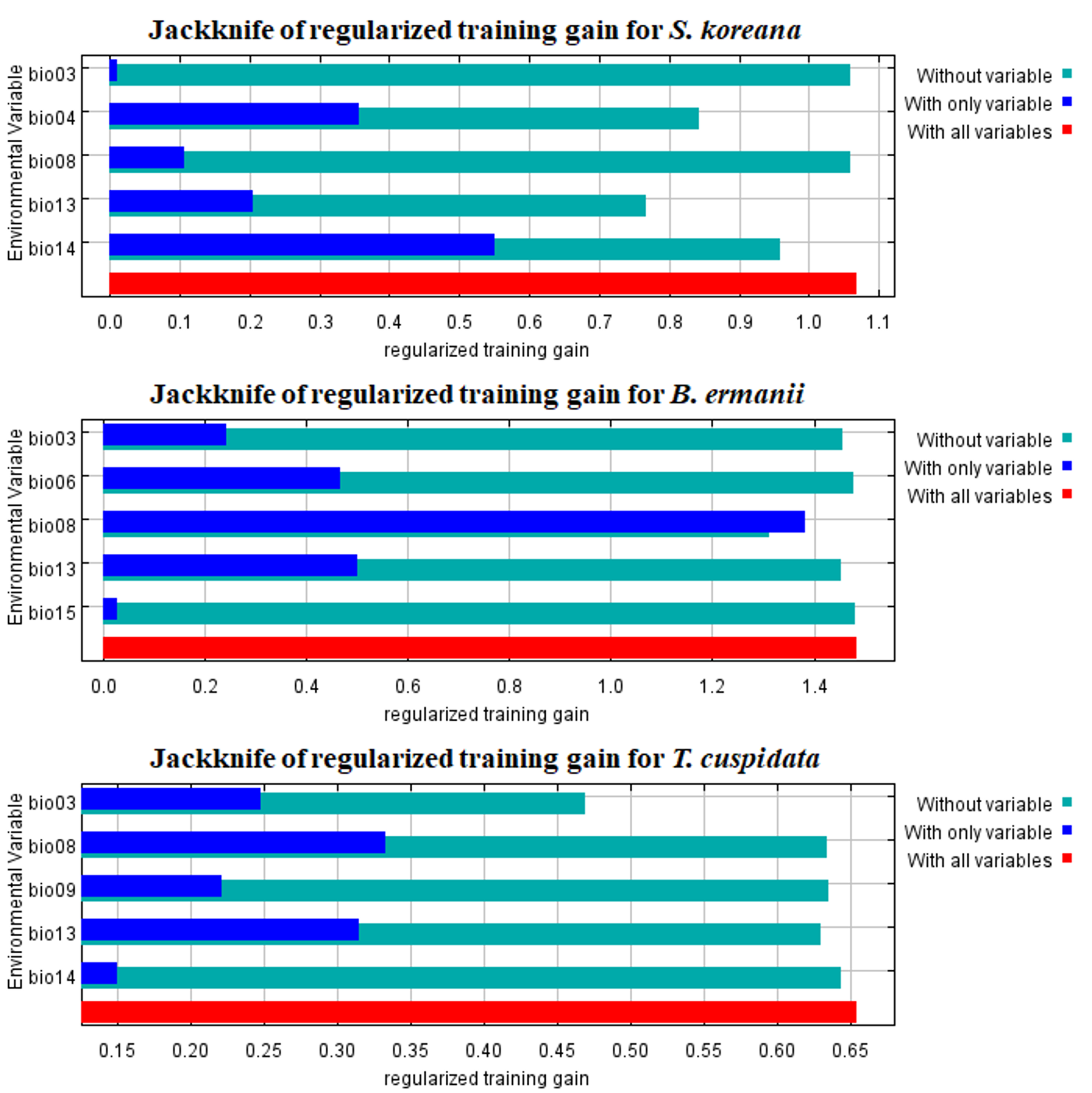

Supplement: S3 Fig — The Jackknife test results show the contribution of each environmental variable to the MaxEnt model’s regularized training gain for Stewartia koreana Nakai ex Rehder (Theaceae) (top), Betula ermanii Cham. (Betulaceae) (middle), and Taxus cuspidata Siebold & Zucc. (Taxaceae) (bottom). The blue bars represent the model’s performance with only one variable, the green bars show the performance without given variable, and the red bars indicate the performance with all variables included. (TIF) [file pone.0316393.s003.tif]

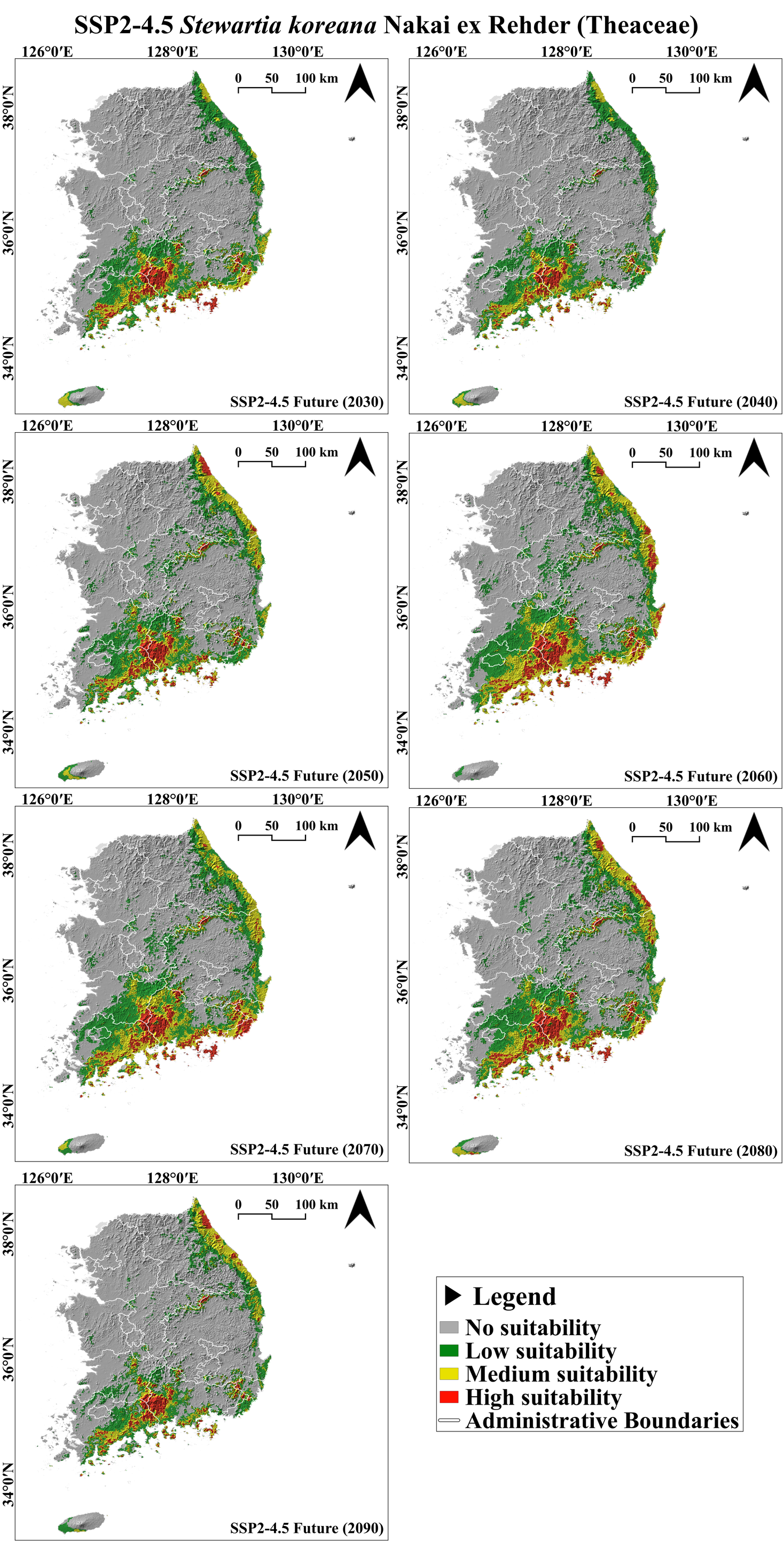

Supplement: S4 Fig — The maps for Stewartia koreana Nakai ex Rehder (Theaceae) illustrate the projected climate suitability areas for the target species in South Korea across seven future time periods (2030, 2040, 2050, 2060, 2070, 2080, and 2090) under the SSP2-4.5 scenario. The suitability is classified into four categories: “No suitability” (gray), “Low suitability” (green), “Medium suitability” (yellow), and “High suitability” (red). (TIF) [file pone.0316393.s004.tif]

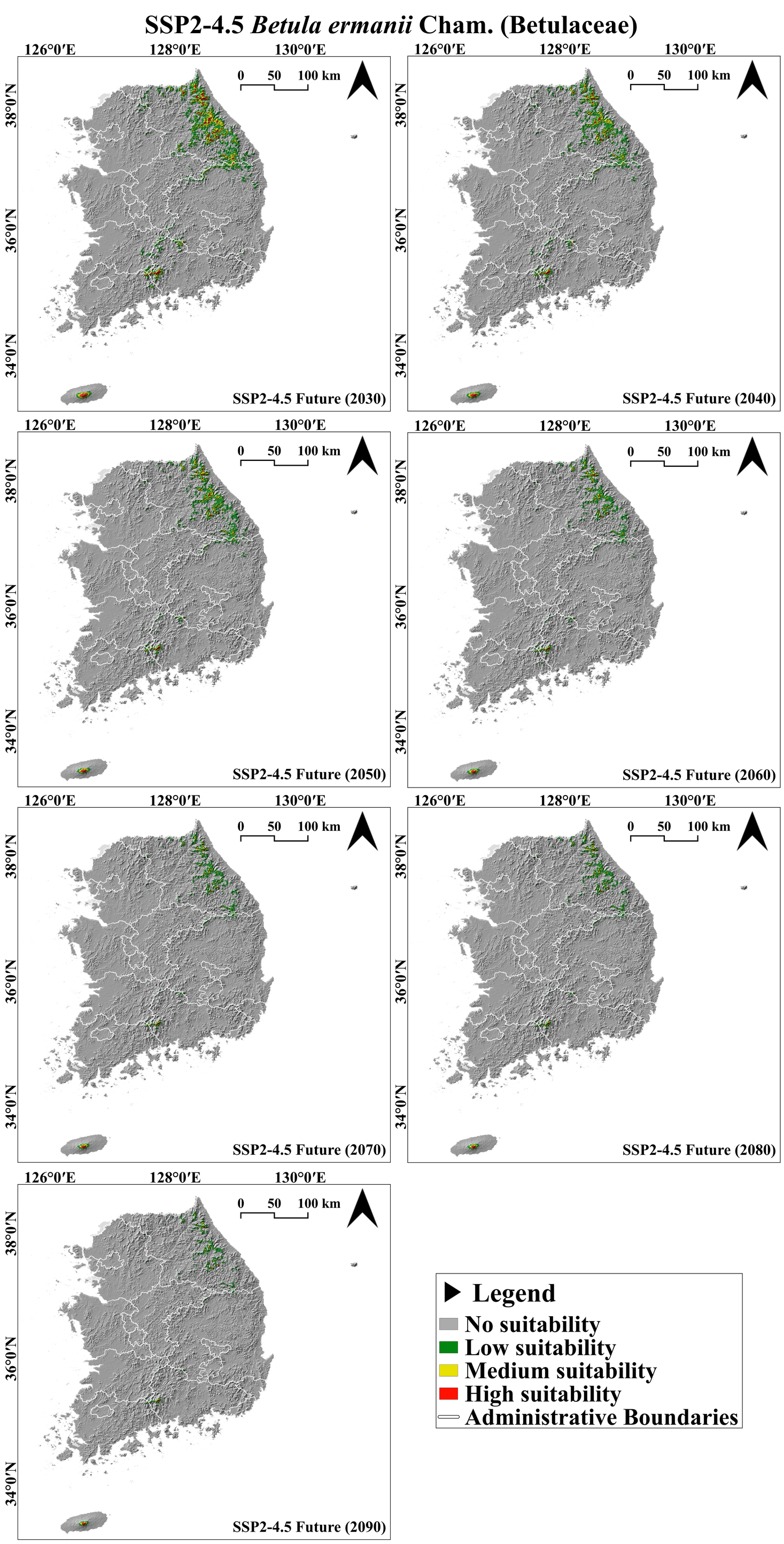

Supplement: S5 Fig — The maps for Betula ermanii Cham. (Betulaceae) illustrate the projected climate suitability areas for the target species in South Korea across seven future time periods (2030, 2040, 2050, 2060, 2070, 2080, and 2090) under the SSP2-4.5 scenario. The suitability is classified into four categories: “No suitability” (gray), “Low suitability” (green), “Medium suitability” (yellow), and “High suitability” (red). (TIF) [file pone.0316393.s005.tif]

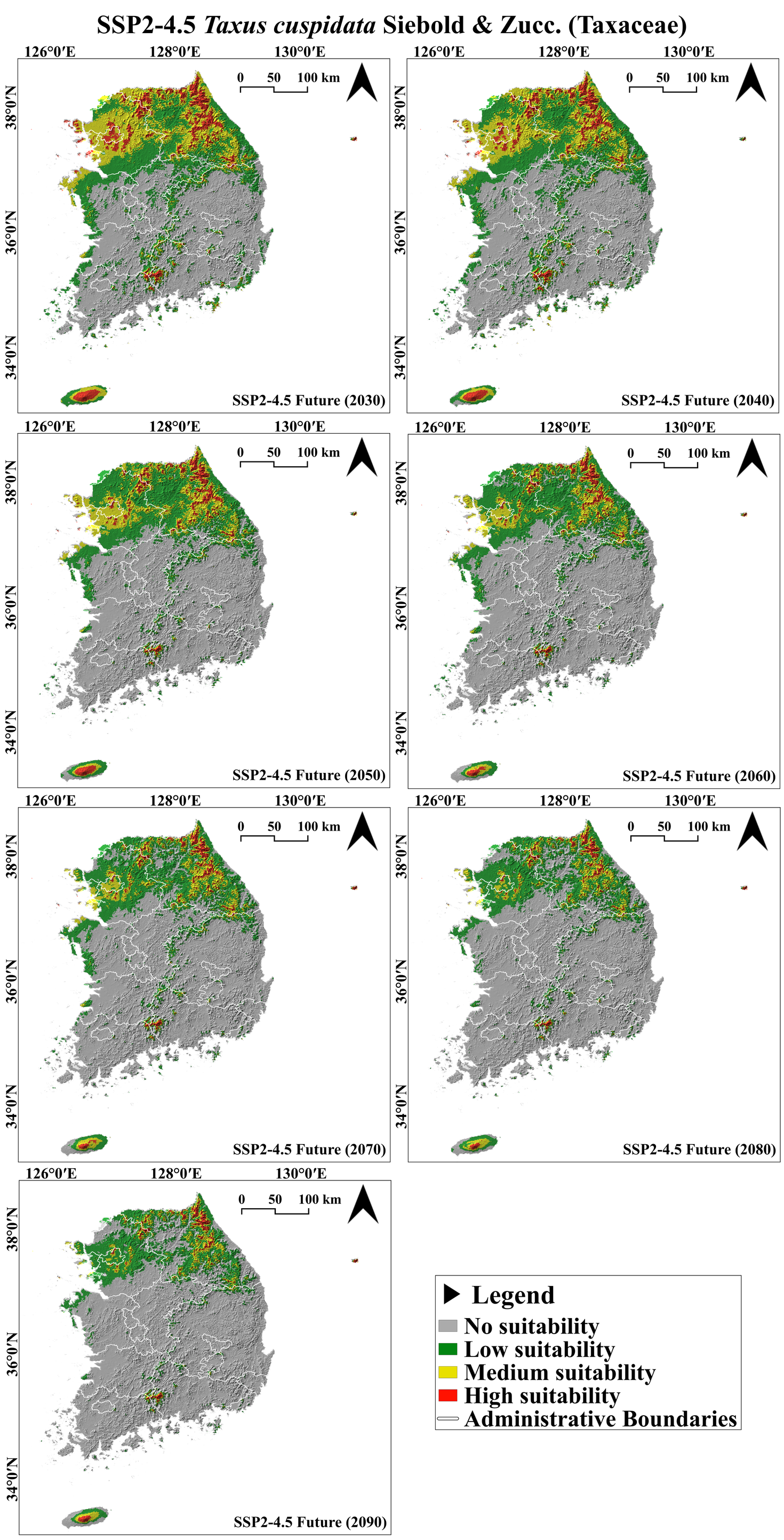

Supplement: S6 Fig — The maps for Taxus cuspidata Siebold & Zucc. (Taxaceae) illustrate the projected climate suitability areas for the target species in South Korea across seven future time periods (2030, 2040, 2050, 2060, 2070, 2080, and 2090) under the SSP2-4.5 scenario. The suitability is classified into four categories: “No suitability” (gray), “Low suitability” (green), “Medium suitability” (yellow), and “High suitability” (red). (TIF) [file pone.0316393.s006.tif]

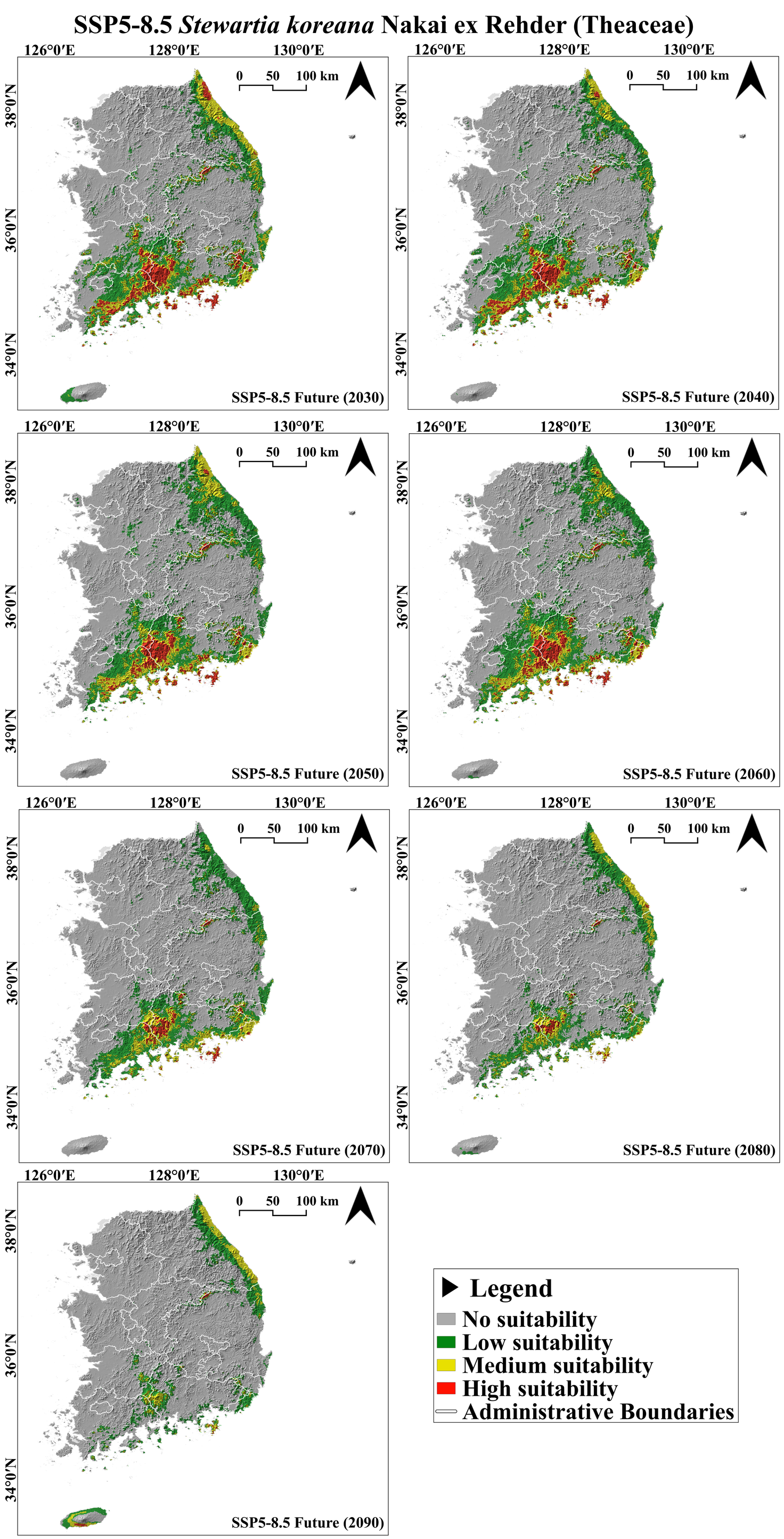

Supplement: S7 Fig — The maps for Stewartia koreana Nakai ex Rehder (Theaceae) illustrate the projected climate suitability areas for the target species in South Korea across seven future time periods (2030, 2040, 2050, 2060, 2070, 2080, and 2090) under the SSP5-8.5 scenario. The suitability is classified into four categories: “No suitability” (gray), “Low suitability” (green), “Medium suitability” (yellow), and “High suitability” (red). (TIF) [file pone.0316393.s007.tif]

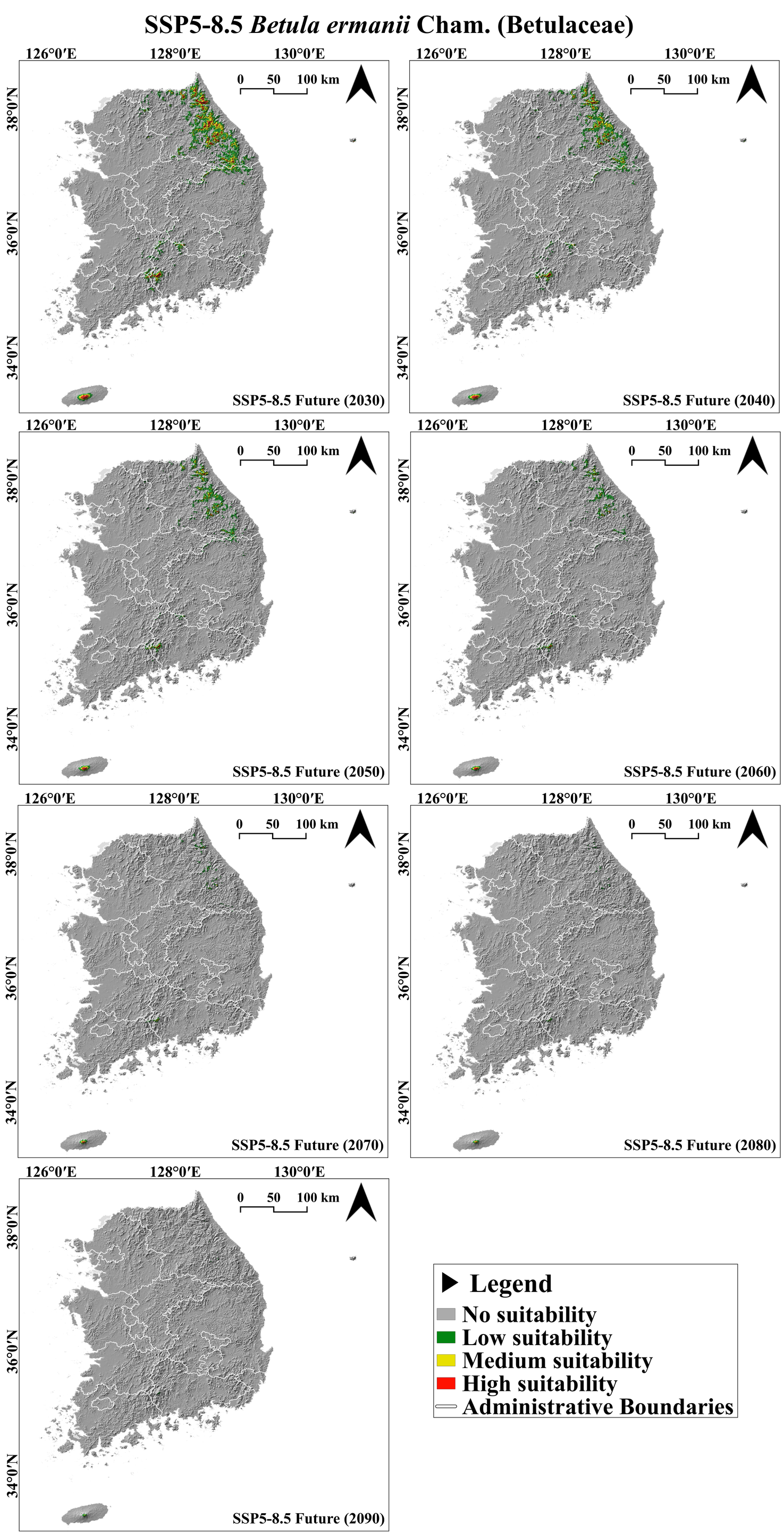

Supplement: S8 Fig — The maps for Betula ermanii Cham. (Betulaceae) illustrate the projected climate suitability areas for the target species in South Korea across seven future time periods (2030, 2040, 2050, 2060, 2070, 2080, and 2090) under the SSP5-8.5 scenario. The suitability is classified into four categories: “No suitability” (gray), “Low suitability” (green), “Medium suitability” (yellow), and “High suitability” (red). (TIF) [file pone.0316393.s008.tif]

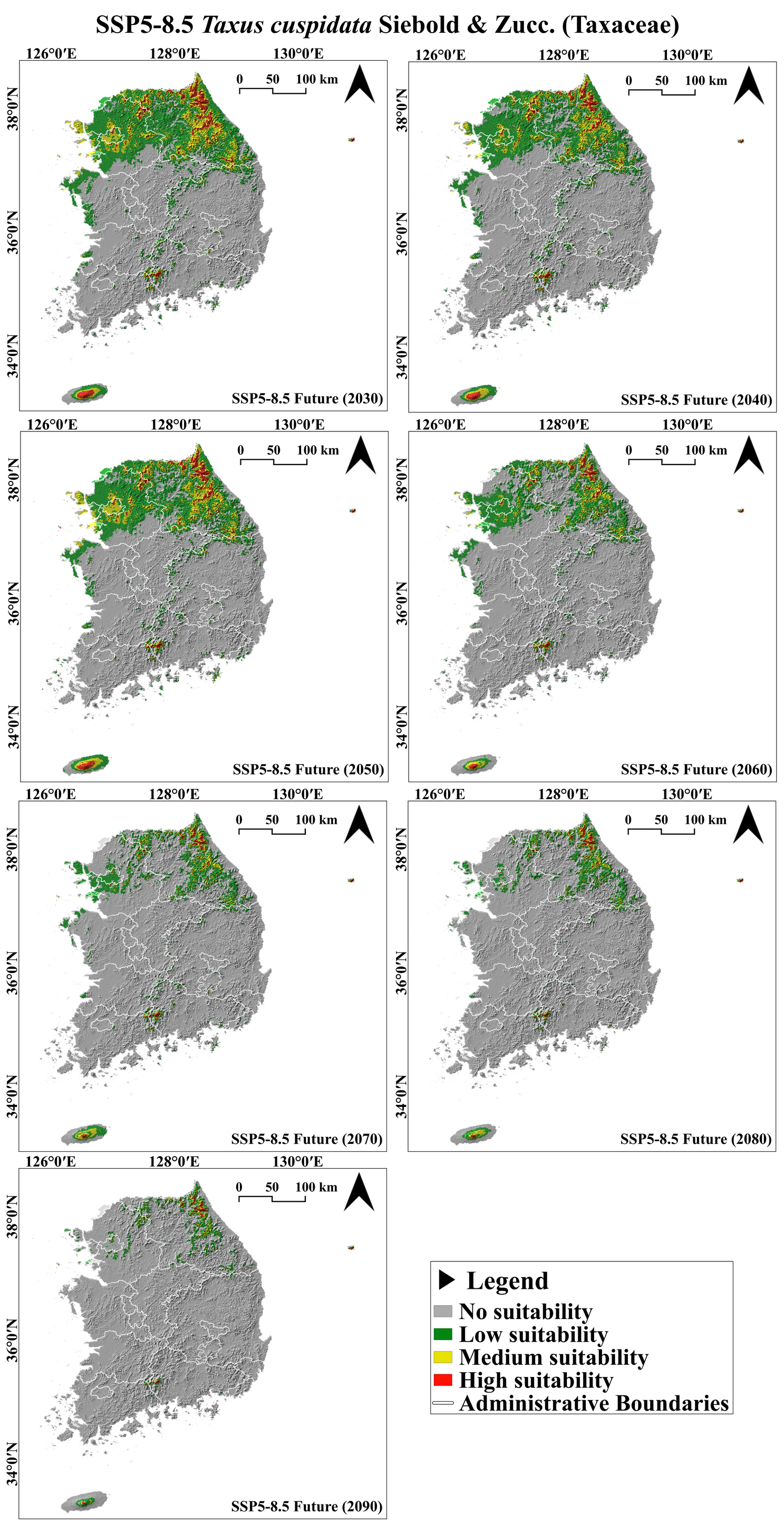

Supplement: S9 Fig — The maps for Taxus cuspidata Siebold & Zucc. (Taxaceae) illustrate the projected climate suitability areas for the target species in South Korea across seven future time periods (2030, 2040, 2050, 2060, 2070, 2080, and 2090) under the SSP5-8.5 scenario. The suitability is classified into four categories: “No suitability” (gray), “Low suitability” (green), “Medium suitability” (yellow), and “High suitability” (red). (TIF) [file pone.0316393.s009.tif]
