# Supplementary material for: Assessment of climate change impact on landscape tree distribution and sustainability in South Korea using MaxEnt-based modeling
Source: PLoS One. 2025 Mar 3;20(3):e0316393. doi: 10.1371/journal.pone.0316393 (PMC11875377; doi:10.1371/journal.pone.0316393)
Supplement: S1 Table — This table presents the bioclimatic variable used in the species distribution modeling for Stewartia koreana Nakai ex Rehder (Theaceae), Betula ermanii Cham. (Betulaceae), and Taxus cuspidata Siebold & Zucc. (Taxaceae). Each variable is listed by its code (e.g., Bio03, Bio04) along with a brief description and the unit of measurement. (DOCX) [file pone.0316393.s010.docx]

**Table S1.** The most impactful bioclimatic variables for *Stewartia koreana* Nakai ex Rehder (Theaceae), *Betula ermanii* Cham. (Betulaceae), and *Taxus cuspidata* Siebold & Zucc. (Taxaceae).

| Species | Code | Description | Unit |
| --- | --- | --- | --- |
| *S. koreana* | Bio03 | Isothermality (bio2 / bio7) (× 100) |  |
|  | Bio04 | Temperature seasonality (standard deviation*100) |  |
|  | Bio08 | Mean temperature of the wettest quarter | ℃ |
|  | Bio13 | Precipitation of the wettest month | mm |
|  | Bio14 | Precipitation of the driest month | mm |
| *B. ermanii* | Bio03 | Isothermality (bio2 / bio7) (× 100) |  |
|  | Bio06 | Min temperature of the coldest month | ℃ |
|  | Bio08 | Mean temperature of the wettest quarter | ℃ |
|  | Bio13 | Precipitation of the wettest month | mm |
|  | Bio15 | Precipitation seasonality (Coefficient of variation) |  |
| *T. cuspidata* | Bio03 | Isothermality (bio2 / bio7) (× 100) |  |
|  | Bio08 | Mean temperature of the wettest quarter | ℃ |
|  | Bio09 | Mean temperature of the driest quarter | ℃ |
|  | Bio13 | Precipitation of the wettest month | mm |
|  | Bio14 | Precipitation of the wettest month | mm |
