# Supplementary material for: Assessment of climate change impact on landscape tree distribution and sustainability in South Korea using MaxEnt-based modeling
Source: PLoS One. 2025 Mar 3;20(3):e0316393. doi: 10.1371/journal.pone.0316393 (PMC11875377; doi:10.1371/journal.pone.0316393)
Supplement: S2 Table — This table shows the percent contribution and permutation importance of key bioclimatic variables used in the species distribution modeling for Stewartia koreana Nakai ex Rehder (Theaceae), Betula ermanii Cham. (Betulaceae), and Taxus cuspidata Siebold & Zucc. (Taxaceae). Percent contribution indicates the extent to which each variable influenced the MaxEnt model’s predictions, while permutation importance reflects the dependence of the model’s accuracy on each variable. (DOCX) [file pone.0316393.s011.docx]

**Table S2.** Percent contribution and permutation importance of each selected bioclimatic variable for *Stewartia koreana* Nakai ex Rehder (Theaceae), *Betula ermanii* Cham. (Betulaceae), and *Taxus cuspidata* Siebold & Zucc. (Taxaceae).

| **Species** | **Code** | **Description** | **Percent**  **contribution** | **Permutation**  **importance** |
| --- | --- | --- | --- | --- |
| *S. koreana* | Bio04 | Temperature seasonality (standard deviation*100) | 34.6 | 43 |
|  | Bio13 | Precipitation of the wettest month | 25 | 38 |
|  | Bio14 | Precipitation of the driest month | 24.9 | 17.6 |
|  | Bio08 | Mean temperature of the wettest quarter | 11.7 | 0.8 |
|  | Bio03 | Isothermality (bio2 / bio7) (× 100) | 3.8 | 0.6 |
| *B. ermanii* | Bio08 | Mean temperature of the wettest quarter | 85.1 | 83 |
|  | Bio13 | Precipitation of the wettest month | 6.7 | 8.1 |
|  | Bio03 | Isothermality (bio2 / bio7) (× 100) | 6.4 | 5.3 |
|  | Bio15 | Precipitation seasonality (Coefficient of variation) | 1.3 | 0.8 |
|  | Bio06 | Min temperature of the coldest month | 0.5 | 2.8 |
| *T. cuspidata* | Bio03 | Isothermality (bio2 / bio7) (× 100) | 37.9 | 52.5 |
|  | Bio08 | Mean temperature of the wettest quarter | 31.3 | 14.4 |
|  | Bio13 | Precipitation of the wettest month | 20.3 | 9.6 |
|  | Bio14 | Precipitation of the wettest month | 6.8 | 5.3 |
|  | Bio09 | Mean temperature of the driest quarter | 3.7 | 18.2 |
